# Application of Nanomaterials in Biomedical Imaging and Cancer Therapy

**DOI:** 10.3390/nano10091700

**Published:** 2020-08-29

**Authors:** Sarkar Siddique, James C. L. Chow

**Affiliations:** 1 Department of Physics, Ryerson University, Toronto, ON M5B 2K3, Canada; sarkar.siddique@ryerson.ca; 2 Radiation Medicine Program, Princess Margaret Cancer Centre, University Health Network, Toronto, ON M5G 1X6, Canada; 3 Department of Radiation Oncology, University of Toronto, Toronto, ON M5T 1P5, Canada

**Keywords:** nanoparticles, application, biomedical imaging, cancer therapy

## Abstract

Nanomaterials, such as nanoparticles, nanorods, nanosphere, nanoshells, and nanostars, are very commonly used in biomedical imaging and cancer therapy. They make excellent drug carriers, imaging contrast agents, photothermal agents, photoacoustic agents, and radiation dose enhancers, among other applications. Recent advances in nanotechnology have led to the use of nanomaterials in many areas of functional imaging, cancer therapy, and synergistic combinational platforms. This review will systematically explore various applications of nanomaterials in biomedical imaging and cancer therapy. The medical imaging modalities include magnetic resonance imaging, computed tomography, positron emission tomography, single photon emission computerized tomography, optical imaging, ultrasound, and photoacoustic imaging. Various cancer therapeutic methods will also be included, including photothermal therapy, photodynamic therapy, chemotherapy, and immunotherapy. This review also covers theranostics, which use the same agent in diagnosis and therapy. This includes recent advances in multimodality imaging, image-guided therapy, and combination therapy. We found that the continuous advances of synthesis and design of novel nanomaterials will enhance the future development of medical imaging and cancer therapy. However, more resources should be available to examine side effects and cell toxicity when using nanomaterials in humans.

## 1. Introduction

In the past 10 years, there have been advances in nanomaterials, such as the development of hundreds of nanoparticles (NPs)-based probes for molecular imaging. The use of NPs has enhanced almost all major imaging techniques, particularly magnetic resonance imaging (MRI), positron emission tomography (PET), and optical imaging. Some of the important milestones are the use of iron oxide NPs in $T_{1}$ weighted and/or $T_{2}$ weighted MRI, the design of radioisotope chelator free (use of radioactive metals that form a stable interaction directly with the surface or core of the NP) particles for PET, and the development of fluorescent NPs such as carbon dots and upconverting NPs [1]. On the other hand, novel types of optical nanoprobes, such as persistent luminescence nanoparticles (PLNPs), are being developed to take advantage of long lasting near-infrared (NIR) luminescence capability [2]. This allows optical imaging without constant excitation and autofluorescence [3].

The latest research and advancement in nanotechnology lead to the development of various NPs for diagnostic and therapeutic applications. Even though clinically, the number of usages of NPs is limited by the complex demands on their pharmacokinetic properties, nanodiagnostics improve the understanding of important physiological principles of various diseases and treatments. On the other hand, NPs are widely used in the clinic for therapeutic purposes. Therapeutic NPs improve the accumulation and release of pharmacologically active agents at the pathological site, which overall, increases therapeutic efficacy and reduces the incidence and intensity of the side effects. NPs hold great promise for integrating diagnostic and therapeutic agents into a single NP for theranostic purposes. A good example would be monitoring biodistribution and target site accumulation, quantifying and visualizing drug release, and longitudinally assessing therapeutic efficacy. Theranostic NPs can be used for personalized nanomedicine-based therapies [4]. Nanoparticles’ intrinsic unique magnetic or optical properties make their application ideal for various imaging modalities. Nanoparticles make excellent contrast agents due to their high sensitivity, small size, and composition. Nanoparticles are often conjugated with suitable targeting ligands on the surface of the particles. Multifunctional NPs can be developed by incorporating various functional materials, and this enables multimodal imaging and therapy simultaneously, also known as theranostics [5].

Although each of the imaging and therapy modalities has improved significantly over the past few years, there are caveats in nanomaterial application that are impeding its application. For example, no single molecular imaging modality can offer all the required data fully characterizing the properties of an administered agent. Each imaging modality has a major shortcoming, such as MRI has high-resolution but low sensitivity, optical techniques have limited tissue penetration, and radioisotope imaging techniques have relatively poor resolution but high sensitivity. Combining multiple imaging techniques can enable these applications to complement one another, and a multimodal imaging agent becomes the key to enhancing those imaging systems [6].

This review analyzed the different roles of nanomaterials, such as contrast agent and dose enhancer, in biomedical imaging and cancer therapy. Moreover, the review discussed the underlying mechanisms of nanomaterials including physical, chemical, and biological mechanisms. Some new applications of nanomaterials as theranostic agents are explored. Through a thorough understanding of the recent advances in nanomaterial application in biomedical imaging and cancer therapy, we identified new directions for the optimization and clinical transformation of nanomaterials.

## 2. Medical Imaging

Medical imaging has improved significantly in recent decades and allows us to precisely obtain anatomical information via different modalities. Nanoparticles play a significant part in medical imaging, as discussed below:

### 2.1. Magnetic Resonance Imaging

Magnetic resonance imaging is a noninvasive imaging technique that can provide multiparametric and comprehensive information [7]. In 1980s, magnetic resonance imaging was introduced, and it revolutionized modern medical imaging technology. It quickly became one of the best paraclinical diagnostic and monitoring tools available [8]. In 2015, an estimate of 17 million MRI examinations were performed in the United States with the use of contrast agents. The contrast agent enhances the image and plays an important role in MRI. An ideal contrast agent should be injected and eliminated from the body without any adverse effects; however, many of the current contrast agents show side effects such as allergic reactions, nephrotoxicity, gadolinium deposition, and physiologic reactions [9]. Recent advances in NPs show their potential to be used as a contrast agent in MRI and minimize many of the side effects. 

#### 2.1.1. Gadolinium (Gd)

Gadolinium-based contrast agents have been used for diagnostic MRI for the last 30 years and have continued to be studied for more functional and improved applications. Recent advances in Gd show that when it is exposed to $Zn^{+2}$ ions, they have increased $r_{1}$ relaxivity [10]. This characteristic has multiple advantages in various applications. Since $Zn^{+2}$ ions are important in the biological process involving enzyme catalyst reaction, they can be used as a biomarker for insulin secretion in β-cells. The prostate contains a high volume of $Zn^{+2}$, which can be used to enhance the image contrast in MRI. Collagen is dysregulated in the diseased cell or cancer cells, and excess production of collagen is seen in common liver conditions such as alcohol and/or drug abuse. Magnetic resonance imaging can be used to detect excess collagen with Gd NPs-based contrast agents. With increased $r_{1}$ relaxivity, Gd can covalently attach to a larger molecule, which does not involve water exchange. Multiple Gd can be attached to a target molecule, along with enhanced permeation and retention effects, which increases MRI contrast significantly [11]. Gadolinium can also be used as a contrast agent and carrier for IL-13 liposome to bypass the blood–brain barrier and use the interleukin-13 receptor as a targeting moiety in the detection of glioma [12].

Dendrimers have great potential in nanomedical imaging and MRI applications. They have very adventitious properties such as their rigidity, low polydispersity, and ease of surface modification. Some applications of dendrimers in MRI are cell tracking, lymph node imaging, blood pool imaging, and tumour-targeted theranostic. Gadolinium is a paramagnetic agent with one of the highest relaxivities due to the high rotational correlation time of the large dendrimer molecules. The relaxivity per Gd (III) ion of the dendrimer is enhanced up to six-fold compared to that of a single Gd (III) chelate. Dendrimer-based Gd contrast agents provide excellent contrast on 3D time of flight MR angiograms. Target-specific bindings of Gd dendrimer can significantly enhance cellular uptake, for example, a cyclic peptide specifically binds to fibrin fibronectin in conjugation with the Gd dendrimer. In one study, Arg-Gly-Asp-Phe-Lys (mpa)(RGD) peptide complex was used as a targeting moiety in combination with a multimodal Gd dendrimer contrast agent and gold nanoparticles (Au NPs) as carriers. It was able to visualize alpha V beta 3-integrin overexpressing tumour cells on both computed tomography (CT) and MRI. Targeted dendrimers can also be used as a therapeutic agent. In neutron capture therapy, dendrimers are irradiated with an external neutron beam; then, the dendrimer-bound Gd generates auger electrons that are highly cytotoxic to tumour cells. This method requires a high accumulation of Gd in the target cell and has been tested on SHIN3 ovarian carcinomas. Recently, Gd-based dendrimers have been even further optimized and provided us with Gd-17, which is based on a poly-L-Lysine dendrimer scaffold, glycodendrimers, and self-assembled dendritic-like NPs. In one study, a manganese-chelating hexametric dendrimer containing six tyrosine-derived [Mn(EDTA)($H_{2}$O)](-2) moieties exhibited relaxivities ranging from 8.2 to 3.8 nM^−1^ s^−1^ under 0.47 to 11.7 T, which was six-fold higher on a per molecule basis compared to a single moiety. This study also showed in a targeted manganese dendrimer, when conjugated with antibody-specific malondialdehyde lysine epitopes, observed enhancement larger than 60% compared to the untargeted counterpart. Along with MRI, manganese dendrimer can also be used as a dual-mode agent for CT-MRI [13]. Another study showed gadolinium oxide with diethylene glycol polymer and magneto liposome NPs in Hepa 1–6 cell lines could be used as a positive MRI contrast agent and marker for cell tracking [14]. Gadolinium chelates have been used in clinical use for a long time and were primarily considered safe. However, recent studies showed an association between a clinically approved Gd-based contrast agent and the development of nephrogenic systemic fibrosis [15]. A few other studies showed a Gd-based contrast agent can potentially result in Gd deposition in human bone and brain tissue, even in the presence of normal renal function [16,17].

#### 2.1.2. Super Paramagnetic Iron Oxide Nano Particles (SPIONs)

Magnetic resonance imaging contrast agents are classified as either $T_{1}$ (positive) or $T_{2}$ (negative). Radiologists primarily prefer the $T_{1}$ positive contrast due to the ease of distinguishability of internal bleeding and air tissue boundaries. Gadolinium-based contrast agents are $T_{1}$ contrast agents, and even though they provide good image enhancement, they have a small risk of adverse side effects. Superparamagnetic iron oxide nanoparticles are a good alternative to Gd. They have a hydrodynamic diameter ranging from 1 to 100 nm. In general, large SPIONs function as $T_{2}$ contrast agents, whereas small SPIONs function as $T_{1}$ [18]. Superparamagnetic iron oxide nanoparticles are particles formed by small crystals of iron oxide, and the coating can be made of organic compounds. Three different types of iron oxide may make up the SPIONs core: hematite (α-$Fe_{2}O_{3}$), magnetite ($Fe_{3}O_{4}$), and maghemite (γ-$Fe_{2}O_{3}$). Superparamagnetic iron oxide nanoparticles can be conjugated to a variety of particles, such as antibodies, and can also be used as a drug carrier in cancer therapy [19]. A study was conducted to observe the efficiency and viability of SPIONs’ tracking ability of stem cells. In one study, FereTRack Direct, a SPION was used in various stem cells. Magnetic resonance imaging was used to monitor the homing-labelled stem cell and cytotoxicity was observed. The study results showed that it was effective at tracking the stem cells in glioma-bearing mice [20]. Clinical translation was greatly increased with the improvement in the delivery system and the ability to track and monitor injected cells. Superparamagnetic iron oxide nanoparticles can be used to label cells and can easily be monitored using MRI [21]. Clustering SPIONs into a raspberry shape within a polymeric envelope outputs a vastly superior image contrast. A study was conducted to observe the effect of increased transverse relaxivity in ultra-small superparamagnetic iron oxide NPs used in MRI contrast agents. Spherical magnetic iron oxide NPs with 12 ± 2 nm size exhibited having superior $T_{2}$ relaxation rate and high relaxivities. Due to strong relaxation properties of the NPs before and after NP administration, MRI analysis shows the clear distinguished signal intensity of specific organ imaging, tumour imaging, and whole-body imaging [22]. Superparamagnetic iron oxide nanoparticles have gained considerable attention as a $T_{2}$ contrast agent due to their unique magnetic properties. However, several SPIONs have recently been discontinued due to a variety of reasons, such as poor contrast enhancement when compared with Gd-based contrast agents. Gadolinium-based contrast agents still need to be investigated thoroughly due to toxicity concerns [23].

Molecular imaging combines multidisciplinary knowledge and expertise from several disciplines such as medical physics, imaging technology, molecular biology, bioinformatics, and mathematics. Molecular imaging allows the study of biochemical processes of disease without disturbing the integrity of the living subject (noninvasive imaging). Magnetic resonance imaging is well-suited to molecular imaging due to its inherent noninvasive properties and excellent spatial resolution. Inflammation is a process that prepares the ground for tissue healing. Due to the involvement of inflammation in the pathogenesis of various human conditions including infection, ischemia, atherosclerosis, and formation of tumour metastasis, monitoring the inflammatory process is clinically important. Misdirected leukocytes may damage healthy tissue by inducing inflammation, where molecular methods and markers can monitor such processes. Target inflammatory cells can be tagged with SPIONs through the internalization of the NPs. Superparamagnetic iron oxide nanoparticles-tagged macrophages can invade tissues through inflammatory processes. In one study, this method was tested on a model of inflammation in the central nervous system. Upon internalization of SPIONs, microglial cells were detected by MRI. In tumour targeting and imaging, macromolecular antibodies with cancer cell surface receptors are the most favoured targeting moieties for the functionalization of NPs due to their high specificity. A well-known tumour target, human epidermal growth factor receptor 2 (Her-2/neu receptor), was attached to poly (amino acid)-coated NPs, where approximately eight Her-2/neu antibodies attached per particle. $T_{2}\;$weighted MRI confirmed that the functionalized NPs could specifically target the Her-2/neu receptors on the cell surface. Drawbacks to antibody-targeted NPs are their large hydrodynamic size and poor diffusion through biological barriers. Nanoparticles functionalized with single-chain antibody fragments (scFvs) can help to solve that problem, since they are smaller in size and can more easily cross the biological membrane. In the case of breast cancer, more than half of human breast cancers express receptors for luteinizing hormone-releasing hormone (LHRH). Nanoparticles functionalized with LHRH can selectively accumulate in primary tumour cells and metastatic cells. Some of the tumour cells overexpress transferrin receptors (TfRs), so Tf-SPIONs can be used for specific labelling and detection of gliosarcoma and breast carcinoma. Folate receptors are generally overexpressed in cancerous tissues. Folate molecules as a targeting ligand can be grafted on SPIONs with different coatings such as PEG, Dextran, and 2-carboxyethyl phosphoric acid target-specific binding [24].

#### 2.1.3. Carbon

##### Carbon-13 (*^13^C*) 

Carbon-13 (*^13^C*) MRI is a very useful metabolic imaging technique because carbon is the backbone for all organic molecules. It can observe a wide range of biological processes relevant to human disease. The MRI signal of carbon-13 is very low due to its natural abundance (1.1%). However, hyperpolarization of *^13^C* increases the signal significantly (more than 10,000-fold) and allows nonradioactive, real-time, safe, and pathway-specific investigation of dynamic metabolism and physiologic processes, which were previously not possible in imaging. The most used hyperpolarized carbon probe is [1-*^13^C*] pyruvate. Its polarization reached up to 50% polarization level in current clinical polarizers and it has a long $T_{1}$ relaxation (approximately 67 sec in solution at 3.0 T). Pyruvate has been used to study metabolism in a variety of diseases such as ischemia inflammation and cancer. Pyruvate is also useful for monitoring early anticancer therapies and study energy metabolism involving cardiovascular disease. It can also be used to investigate metabolic changes related to hypoxia and oxidative stress [25]. Pyruvate can be used as a tool to predict cancer progression, characterize cancer biology, and be used as a biological marker [26]. The Warburg effect is where cancer cells exhibit elevated levels of glycolysis and lactic acid fermentation. Hyperpolarized pyruvate can be used to quantify the flux. Lactate dehydrogenase-mediated conversion of pyruvate to lactate is elevated in malignant cells as a result of the Warburg effect. The high concentration of glutathione, which correlates to the increased reduction in 1-*^13^C* dehydroascorbate to 1-*^13^C* vitamin C, can be associated with malignancy, and can be used as a detection tool [27]. A NP-based pyruvate biosensor was developed that can detect total pyruvate level in sera [28].

##### Nanodiamonds

Nanodiamond is a nontoxic substrate that can be used for drug delivery and cellular tracking (fluorescent marker). The Overhauser effect is a proton–electron polarization transfer technique that can enable high contrast MRI of nanodiamond in water at room temperature and in an ultra-low magnetic field. Magnetic resonance imaging cannot efficiently detect nanodiamond directly due to low abundance and the small gyromagnetic ratio of spin ½ *^13^C* nuclei, which compromise the carbon lattice. At ultra-low magnetic field, efficient Overhauser polarization transfer between electronic and nuclear spins in a compatible radiofrequency is possible. Radiofrequency pulsing of the electron paramagnetic resonance transition between MRI signals continually transfers spin polarization from the paramagnetic centers at the surface of nanodiamond to *^1^H* nuclei in the surrounding water. Therefore, the presence of nanodiamond in water produces an enhancement in the *^1^H* MRI signal, which can produce images with contrast sensitivity to nanodiamond concentrations [29].

##### Carbon Nanotubes

Carbon nanotubes can be synthesized single-walled or multiwalled commercially and have diameters in the nm range and length in the µm range. Since carbon nanotubes can easily be internalized by living cells, they are expected to have a wide range of applications in biomedicine such as imaging and therapy. However, carbon nanotubes are insoluble in most solvents. Therefore, noncovalent coating of amphiphilic molecules or functionalization of various chemical groups on the nanotube surface are carried out to make the nanotubes soluble in biologically compatible buffers. The unique electromagnetic property of carbon nanotubes makes them highly sensitive in various imaging modalities such as photoacoustic molecular imaging and NIR imaging [30]. Photoacoustic imaging allows higher resolution and deeper imaging depth than optical imaging. It is found that a single-walled carbon nanotube conjugated with cyclic ArgGly-Asp (RGD) peptides can be used as an effective contrast agent for tumours. A preclinical study showed that eight times the photoacoustic signal in the tumour could be acquired with mice injected with targeted nanotubes compared to mice with nontargeted nanotubes [31]. For NIR imaging, another preclinical study showed that single-walled carbon nanotubes with sodium cholate could be used as in vivo imaging agents to produce high-resolution imaging with deep tissue penetration and low autofluorescence in the NIR region beyond 1 µm [32].

##### Graphene

Graphene is a single layer of carbon atoms arranged in a 2D honeycomb lattice. Due to its excellent physicochemical, surface engineering, and biological properties such as small hydrodynamic size, low toxicity, and biocompatibility, graphene-based nanomaterials, namely, graphene–dye conjugates, graphene–antibody conjugates, graphene–NP composites, and graphene quantum dots can act as an in vitro and in vivo imaging agent for molecular imaging [33]. In an in vitro and in vivo study, carboxylated photoluminescent graphene nanodots were synthesized for photoluminescent experiments. It was found that the nanodots could enhance the visualization of tumour in mice and therefore, was proved to be an effective optical imaging agent for detecting cancer in deep tissue noninvasively [34].

#### 2.1.4. Manganese (*Mn)*

Manganese-based contrast agents have good biocompatibility and ideal characteristics for MRI, such as the short circulation time of Mn(II) ion chelate in the $T_{1}$ weighted image. Manganese oxide NPs have negligible toxicity and good $T_{1}$ weighted contrast effects. If manganese oxide NPs are retained by the reticuloendothelial system and stored up in the liver and spleen, it will lead to $Mn^{2+}$ induced toxicity. Pegylated bis-phosphonate dendrons are attached to the surface of the manganese oxide NPs, which can solve the problem. This improves colloidal stability, excretion ability, and relaxation performance. Manganese oxide NPs with a hydrodynamic diameter of 13.4 ± 1.6 nm will eventually be discharged through the hepatobiliary pathway as feces or urinary excretion. Polyethylene glycol coating also has a high potential to reduce toxicity with manganese oxide NPs [35].

#### 2.1.5. Silicon (*^29^Si*)

Hyperpolarized silicon particles can be used in MRI applications. Large silicon particles with an average size of 2.2 µm generally have larger polarization than NPs. However, a recent study showed that a much smaller silicon-29 particle (APS = 55 ± 12 nm) can be hyperpolarized. For MRI application, a silicon-based contrast agent can be produced by incorporating transition metal ions into a particle’s body. This contrast agent shortens the nuclear spin-lattice relaxation time ($T_{1}$) of the protons of nearby tissues, and ultimately, amplifies the signal in $T_{1}$ weighted proton imaging. Direct detection of the silicon signal is not possible due to its low sensitivity of *^29^ Si* nuclei, which leads to long acquisition times. However, this limitation can be solved via hyperpolarization. Utilizing this technique, the imaging window span lasts around 60–120 s, which allows rapid enzymatic reactions and anaerobic metabolism to be studied and further be used to characterize the pathology of the tissue. One of the advantages of using a silicon-based contrast agent is its versatility of surface chemistry; the attachment of functional organic molecules on the surface of the particles does not significantly reduce any of the desired nuclear magnetic resonance properties [36].

#### 2.1.6. Peptide

Atherosclerosis contributes to cardiovascular disease and is the leading cause of morbidity and mortality in the United States. Atherosclerosis is characterized as a chronic and inflammatory disease. Early detection of unstable plaques improves treatment success rate significantly. Magnetic resonance imaging is an important imaging modality for cases such as this, due to its ability to image and characterize the blood vessel wall and plaque in a noninvasive manner and without any ionizing radiation. Peptide-based NPs are useful for enhancing MRI images due to their biodegradable properties and inherent biocompatibility. Super molecular peptide amphiphile micelles can be used to target unstable atherosclerotic plaques displaying microthrombi. The peptide amphiphile micelles can be functionalized using two types of amphiphilic molecules containing Gd chelator. This target-specific NP compound enhances the image and detection probability. It can be used in dual optical imaging-MRI [37].

### 2.2. Computed Tomography

Computed tomography works by making use of an x-ray source and a detector array to form images. It has been widely used in clinical imaging for a long time and can produce an image with high spatial and temporal resolution. It can provide 3D anatomical information of specific tissues and organs such as the cardiovascular track, gastrointestinal track, liver, and lung noninvasively. One drawback to CT is that it lacks sensitivity toward contrast agents, where other modalities such as MRI shine. However, there are still few promising contrast agents available for CT [38].

#### 2.2.1. Gold Nanoparticles

Gold nanoparticles have unique x-ray attenuation properties and easy surface modification. Au NPs can be functionalized with glucosamine to be an effective contrast agent [39]. Gold nanoparticles have a high x-ray absorption coefficient and can specifically image tumours using CT with an enhanced permeability and retention effect (EPR). In a breast cancer experiment, Au NPs were conjugated with PEG chains and tumour biomarkers (human epidermal growth factor 2). They were able to provide an enhanced image in CT due to their specific targeting ability [40]. A mesenchymal stem cell is a type of adult stem cell that has high potential in cellular-based regenerative therapy and is able to treat various medical conditions such as autoimmune, neurodegenerative, and cardiovascular disease. They can also be used to repair cartilage and bones. Their most adventitious property is being able to migrate into different tissue, and monitoring this migration is very beneficial for studying metastases. A study was done to observe such migration of mesenchymal cells using Au NPs as a marker, and a micro CT was used to obtain movement information from the Au NP marker [41]. The study observed the comparison between porous and solid Au NPs as a contrast agent and their effect on the liver and kidney. Porous Au NPs show brighter contrast of 45 HU, where solid AuNPs show almost half less (26 HU). Computed tomography scans of porous Au NPs show significantly enhanced contrast as compared to solid Au NPs [42]. A new approach to Au NP-based contrast agents for CT was developed, where they encapsulated biodegradable poly-di (carboxylatophenoxy) phosphazene into gold nanospheres. They can function as a contrast agent, then, subsequently break down into harmless by-products, and the Au NPs can be released through excretion. The CT image shows that these contrast agents can enhance the image significantly and produce a strong contrast image [43].

#### 2.2.2. Iodine (^131^I)

Iodine-based polymer iodine NP contrast agents were introduced for high vascular contrast and tumour loading. They have low cost and their organic structure provides biodegradation and clearance compared to many metal NPs. They are also very small (~20 nm) in size, which provides better tumour penetration compared to larger NPs. The contrast agents have long blood half-life (40 h) that provides better tumour uptake and clearance from the liver when compared to Au NPs. The agents also efficiently accumulate in tumours and provide high contrast vascular tumour imaging [44]. In the imaging of thyroid diseases and radionuclide therapy, iodine has been routinely used due to its high affinity for thyroid and relatively long half-life (8.01 days). It also has other adventitious properties, such as gamma emission that can be used for SPECT imaging and beta minus decay, which can be used for therapeutic purposes. Iodine-labelled glioma targeting ligands such as chlorotoxin have high potential in targeted SPECT imaging and radionuclide therapy of glioma. A study was carried out to functionalize polyethylenimine (PEI)-entrapped Au NPs, which were PEGylated and combined with targeting peptide BmK, and used in CT for targeted CT/SPECT imaging and radionuclide therapy of glioma [45]. The regional lymph node is one of the most frequent sites of early carcinoma metastasis. There was a study to develop a sentinel lymph node tracer consisting of iodine and docetaxel. The results of the study showed that it can simultaneously perform sentinel lymph node CT and locoregional chemotherapy of the draining lymphatic system [46]. In functional imaging of tumours, simultaneous imaging of multiple contrast agents is useful due to simultaneous visualization of multiple targets that allow observation of cancer progression and its development. Iodine and Gd have a previous record of clinical use as image enhancing agents. A study was carried out to see the viability of them to be used as contrast agents in both dual-energy micro CT with energy integrating detectors and photon-counting detector-based spectral micro CT. The experimental results showed that the contrast agents provided enhanced images. The photon-counting detector provided a lower background signal, a better simultaneous visualization of tumour vasculature, and an intratumoural distribution pattern of NPs compared to dual-energy micro CT with energy integrating detectors [47].

#### 2.2.3. Bismuth

In a study, a hybrid cluster was synthesized using PEG 2000-DSPE. It contained hydrophobic bismuth ($Bi_{2}S_{3}$) NPs and quantum dots, and could be used as a contrast enhancer for combined CT/fluorescence imaging. The cluster produced contrast enhancement in CT imaging of the liver and spleen after 30 min and lasted for more than 4 h. The experimental results showed that the probe had good biocompatibility and did not disrupt normal organ function [48].

### 2.3. Positron Emission Tomography

Positron emission tomography (PET) is a nuclear medicine imaging technique. It uses radiotracers to produce images of radionuclide distribution. These tracers can provide information on biological pathways via a noninvasive method [49].

#### 2.3.1. Gold Nanoparticles

Gold nanoparticles are commonly used in PET. Visualization of dendritic cell migration is possible with the recent development in highly sensitive, biocompatible, and stable imaging agents. Tracking dendritic cell migration is important in dendritic-based immunotherapy. Novel radioiodine-124-labelled tannic acid gold core–shell NPs were introduced for dendritic cell labelling and tracking using PET imaging. This nanoplatform had good labelling efficiency, high radiosensitivity, and excellent chemical stability. It also had a negligible effect on cell biological function, including phenotype marker expression and proliferation. The experimental results showed they were successful at tracking dendritic cell migration [50]. For early evaluation of photothermal therapy (PTT), a study combined *^18^F—FDG* PET with CT, and diffusion weighted images in tumour-bearing mice using silica gold nanoshells. The NP-treated mice exhibited inhibited tumour growth compared to control mice. Changes in *^18^F—FDG* uptake and apparent diffusion coefficient correlates with tumour survival and it can be used for early evaluation of PTT [51]. For brain tumours, a new *^124^I*-labelled gold nanostar probe using PET was introduced. The experimental results showed that it can potentially reach sub-mm intracranial brain tumour detection, which is superior to any available noninvasive imaging modality [52]. Another study focused on the PEGylated crushed gold shell- radioactive *^124^I*-labelled gold core nanoballs for in vivo imaging application with PET. It has high stability and sensitivity in various pH solutions. Positron emission tomography in combination with Cerenkov luminescent imaging showed tumour lesions at 1 h after an injection of NPs and signals remained visible in tumour lesions up to 24 h [53].

#### 2.3.2. Copper (*^64^Cu)*

Copper sulfide (*CuS*) NPs have noncomplex chemistry, low toxicity, and small particle size, which makes them an ideal imaging agent. Radioactive *[^64^Cu]CuS* NPs can be used as a radiotracer for PET and photothermal ablation therapy using near infra-red NIR laser irradiation. When *[^64^Cu]CuS* NPs are conjugated to RGDfK peptide through PEG linkers, they have targeting ability and significantly higher tumour uptake (8.4 ± 1.4% injected dose/tissue). They can be used as an enhancing PET imaging contrast agent and also be used for theranostic application [54]. A study conjugated natriuretic peptide receptor-binding peptide (targeting entity) with a C-type atrial natriuretic factor was conducted to produce comb *^64^Cu-CANF* NPs. The study showed improved biodistribution profiles and significantly reduced accumulation in both the liver and spleen compared to the control. The study results also demonstrated the potential for it to be a PET imaging agent to detect atherosclerosis progression [55]. For quantitative PET imaging macrophages in tumours, pharmacokinetically optimized *^64^Cu*-labelled polyglucose NPs (Macrin) were developed [56]. Single chain antibodies have high antigen specificity and affinity, modular structure, and fast urinary clearance, which makes them ideal to be used as targeting ligands. An antiprostate membrane antigen, scFv, has site-specific cysteine and was evaluated in the prostate cancer xenograft model by Cu-64 PET imaging. scFv-cys was conjugated to copolymer distearoyl phosphatidyl ethanolamine monomethoxy polyethylene glycol-maleimide that spontaneously assemble into homogeneous multivalent lipid NPs, which enhances tumour accumulation. It exhibited a 2-fold increase in tumour uptake compared to scFv alone. The antiprostate membrane antigen scFv lipid NPs exhibited a 1.6-fold increase in tumour targeting over the nontargeted lipid NPs. This shows its potential to be used in PET as an image enhancer [57].

#### 2.3.3. Other Nanoparticles 

Abundant inflammatory macrophages destructing tissue leads to atherosclerosis, myocardial infarction, and heart failure. Monitoring macrophages in patients can be useful for avoiding or early treating many of these diseases. *^18^F*-Macroflor modified polyglucose NPs have high avidity for macrophages. They have a small size and can excrete renally. Macroflor enriches cardiac and plaque macrophages and they increase the PET signal [58]. A nanoplatform of farnesylthiosalicylate-based copolymer consisting of a poly (oligo (ethylene glycol) methacrylate) hydrophilic block, a poly hydrophobic block, and a poly (4-vinyl benzyl azide) middle block was introduced. The in vivo and vitro nanoplatform inhibits tumour growth and can also serve as a carrier for paclitaxel. It also provides an active azide group for incorporating a PET imaging modality via a facile strategy based on metal-free click chemistry. Its compatible properties allow it to be used for PET image-guided drug delivery [59]. The pH-sensitive pharmaceutical-grade carboxymethylcellulose-based NPs were introduced for white blood cells to be used in PET imaging. *^68^Ga^3+^* was used for labelling, which provides greater spatial resolution and patient convenience for PET over SPECT [60]. Polyphenol and poloxamer self-assembled supramolecular NPs have multiple hydrogen bonding between tannic acid and Pluronic F-127 in combination with hydrophobic reactions of poly (propylene oxide) chains, which can be applied in NIRF and PET imaging. Their excess phenolic hydroxyl groups chelating positron-emitting radionuclide *^89^Zr* function as PET contrast agents. They have good biocompatibility in various cell lines, and in vitro, they do not induce hemolysis [61]. Cerium oxide NPs have unique surface chemistry. Cerium oxide NPs coated with *^89^Zr*, a clinical PET isotope, for PET imaging and in vivo biodistribution were synthesized and showed great potential to be used in PET imaging [62].

### 2.4. Single Photon Emission Computerized Tomography

Single photon emission computerized tomography is a nuclear imaging technique that uses gamma rays to assess biochemical changes and the level of the molecular target within a living subject. For the past few decades, SPECT has been the nuclear imaging technique thanks to *^99m^T_c_* [63].

#### 2.4.1. Gold Nanoparticles

Gold nanoparticles have high potential in SPECT imaging and can be used as a contrast agent [64]. A study showed an alternative method for functionalizing Au NPs with mannose. Technetium (*^99m^T_c_*)-radiolabelled Au NPs functionalized with mannose can track and accumulate in lymph nodes. It can be used as a SPECT contrast agent for lymphatic mapping [65].

#### 2.4.2. Technetium (*^99m^T_c_*)

For the diagnosis of metastases in early-stage cervical cancer patients, a study was carried out to evaluate the accuracy of *^99m^T_c_* SPECT/MRI fusion for selective assessment of nonenlarged sentinel lymph nodes (SLN). The fused datasets of the SPECT and MR images can be used to identify SLN on the MRI with an accurate correlation to the histologic result of each SLN. The results of the study showed that the size and absence of sharp demarcation can be used to noninvasively assess the presence of metastasis in cervical cancer patients without enlarged lymph nodes [66]. *^99m^T_c_* can be used as a targeting agent in SPECT for mapping SLN. *^99m^T_c_* in conjunction with SPECT-MRI has been used for SLN mapping in preoperative assessment of SLN metastases in the early-stage cervical cancer in women [67]. Single photon emission computerized tomography following 2D planer lymphoscintigraphy in conjunction with *^99m^T_c_* NP can be used for dynamic sentinel lymph node biopsy in penile cancer patients. Single photon emission computerized tomography and *^99m^T_c_* improved the rate of detection of true tracer acid lymph nodes and delineated their precise anatomic localization in drainage basins [68]. With the help of *^99m^T_c_* nanocolloid tracer, lymph drainage mapping with SPECT/CT can be used to select patients with minimal risk of contralateral nodal failure for unilateral elective nodal irradiation in head-and-neck squamous cell carcinoma patients [69]. Sentinel lymph node biopsy after intertumoural injection of *^99m^T_c_*-labelled nanocolloid with imaging of scintigraphy and SPECT/CT in renal tumours is feasible. However, the nondetection rate is high [70].

### 2.5. Optical Imaging

Noninvasive optical imaging can visualize various classes of structures involved in autophagy at macroscopic and microscopic dynamic levels. Optical imaging is involved in fluorescence, chemiluminescence, and Raman imaging, which can obtain noninvasive 2D or multidimensional image data at both the macro and micro scale. Fluorescence imaging provides intuitive results, is less time consuming, and can more easily be interpreted than other methods. That is why it is widely preferred by researchers and is used in biomedical imaging application [71].

#### 2.5.1. Fluorescence

In biological study, fluorescent NPs are generally used to localize molecule or highlight processes in living organisms or cell culture. For fluorescence imaging, the excitation and absorption wavelengths should be in the NIR optical window to allow good signal detection. In general, 700–750 nm excitation and 750–800 nm emission wavelengths are found to be the optimal range [72]. Although fluorescence has been used for a long time in biomarker analysis, immunoassays, and diagnostic imaging, it has several shortcomings such as wavelength range, photobleaching, and fluorescence self-quenching. Fluorescent NPs can mitigate some of the shortcomings as they often contain multiple fluorophore entities, which leads to increased photoluminescent emission. Their encapsulation into the particle provides improved stability, reduced photobleaching, and reduced toxicity [73]. Fluorescence imaging in the NIR II window using organic fluorophores has great advantages, but has a few shortcomings, such as relatively low fluorescence quantum yield (less than 2%). There was a study to develop a system with organic NPs (L1013 NPs) with a high fluorescence quantum yield of 9.9%. This was able to noninvasively visualize real-time mouse hindlimb and vessels under a very low power density and short exposure time. It was also able to localize tumour pathology with a tumour to normal tissue ratio of 11.7 ± 1.3. The study results showed its great potential to be used in optical imaging application [74]. The NIR IIb (1500–1700 nm) window is ideal for deep tissue optical imaging, but it faces the same general NIR issues such as lack of bright and biocompatible probes. Cubic phase (α-phase) erbium-based rare earth NPs were introduced to be used in the NIR IIb window. It was functionalized with a cross-linked hydrophilic polymer layer that was attached to the anti-PF-L1 antibody for molecular imaging of PD-L1 in a mouse model of colon cancer. It achieved a tumour to normal tissue signal ratio of ~40%. It had a luminescence lifetime of ~4.6 ms that enabled simultaneous imaging of the nanocomplex. Its cross-linked functionalized layer facilitated 90% of the nanocomplex excretion within two weeks and showed negligible toxicity in mice [75]. Indocyanine green (ICG) is an FDA-approved dye that has been shown to exhibit NIRII fluorescence. It was used to perform imaging tests of real-time visualization of vascular structures in hindlimb and intracranial regions in vivo. Fluorescence spectra show strong NIR II fluorescence of liposomal ICG. In vivo results show the enhanced performance of liposomal ICG over control for imaging of deep (>4 mm) vascular mimicking structures. It also provided a significantly higher contrast to signal ratio for an extended period which allows visualization of the hindlimb and intracranial vasculature for up to 4 h post injection [76]. In current clinical practices, the recent development of fluorescent probes is very important for cancer diagnosis and surgery. Functionalized fluorescent probes can be used as contrast agents. This allows for real-time visualization of the molecular edge between cancer and adjacent normal tissue. Fluorescence-guided surgery helps the operator decide the tissue spearing margin and generally results in a good surgical outcome. It also reduces costs. Fluorescent Au NPs conjugated with diatriozoic acid and nucleolin targeted A$S_{1411}$; the aptamer can be used as a molecular contrast agent to reveal tumour location in the $CL_{1-5}$ tumour. It can also be used as an enhancer for CT due to its high attenuation. The conjugate has good biocompatibility, high water solubility, strong x-ray attenuation, and visible fluorescence [77]. For triple-negative breast cancer and ovarian cancer, a fluorinated tracer, which enables MRI (*^19^F MRI*), shows potential for repeated imaging sessions due to the use of nonionizing radiation. A fluorous particle is derived from the low molecular weight amphiphilic copolymer. It self-assembles into micelles with a hydrodynamic diameter of 260 nm, and it shows negligible toxicity. Fluorine MRI detects molecular signatures by imaging a fluorinated tracer. In vitro and vivo, it was capable of tracking and monitoring immune cells and cancer cells. Their systemic administration exhibited significant uptake into triple negative breast cancer and ovarian cancer with minimum accumulation in off-target tissue [78]. Unravelling complex neural interactions at multiple scales in the brain is complex and often very difficult. However, optical imaging offers a solution. A fluorescent NPs-based probe, particularly calcium-based NPs, correlate with neuronal activity and can be used to monitor a full array of chemicals in the brain with improved spatial temporal and chemical resolution. This enables mapping of the neurochemical circuit with finer precision [79]. Treatment of inflammatory disorders with glucocorticoids is possible with NPs delivery, but their delivery needs to be controlled and monitored to minimize adverse side effects. In vivo glucocorticoid betamethasone phosphate, in conjunction with NPs and fluorescent dye DY-647, can improve the treatment of inflammation with simultaneous monitoring of the delivery [80].

#### 2.5.2. Quantum Dots

Fluorescence imaging allows for visualization of real-time details at a cellular level. However, long-term imaging is difficult due to the poor light stability of organic fluorescent dyes. Graphene quantum dots are a good alternative solution. They have outstanding optical properties and unique structural features. Graphene quantum dots are loaded on the surface of NPs for example optical magneto ferroferric oxide@polypyrrole core–shell NPs. They have longer metabolic processes in blood. In vivo results show they are capable of monitoring the distribution and metabolism of NPs [81]. Mesoporous silica NPs with graphene quantum dots can simultaneously monitor real-time localization of the doxorubicin (DOX) carrier for proper drug targeting as a fluorescent imaging agent. The nanoplatform shows stable suspension and excitation-dependent photoluminescence. They also show pH and temperature-dependent release behaviour that can be utilized in theranostic applications. Their fluorescence properties make them suitable for optical imaging with minimal cytotoxicity [82]. Quantum dots offer superior optical characteristics compared to organic dyes. Their potential toxicity, however, limits their application. Topical administration can reduce toxicity. In vivo endoscopic imaging of human bladder cancer by topical administration of quantum dots conjugated to anti-CD47 was successful and showed no indication of acute toxicity up to 7 days after installation [83]. The molecular profile of most cancer varies greatly between patients as well as spatially and temporally within a single tumour mass. To account for such variance, multiplexed molecular imaging has high potential. Multiplexed molecular imaging enables multiplexed imaging of large panels of cancer biomarkers. Polymer dots can be used in multiplexed molecular imaging and utilizes semiconducting polymers with strong fluorescence. Studies show a 10-fold enhanced brightness of polymer dots over commercial fluorescent dyes. Quantum dots are fluorescent semiconductor NPs that typically contain group II-VI (e.g., CdSe and CdTe), III-V (e.g., InP and InAs), IV-VI (e.g., PbTe, PbSe), or I-III-VI (e.g., *CuIn*$S_{2}$) elements. They have a narrow and symmetric emission band (~30 nm) that can be tuned precisely by changing the NP sizes and compositions. The broad absorption spectra and large stokes shifts of quantum dots allow simultaneous imaging of multiple types of quantum dots with single wavelength excitation. Quantum dots are also often coupled to a biomolecule for targeted imaging [84]. Lead sulfide quantum dots with 1100 nm emission peaks can be used in NIR fluorescence imaging of cerebral venous thrombosis. This was tested in septic mice and the results showed it to be a useful tool for the evaluation of the pathological state of cerebral blood vessels in septic mice [85]. A study introduced short wavelength infrared region emissive indium arsenide quantum dots. They have high-resolution multicolour imaging, are readily modifiable, provide deep penetration, and have fast acquisition speed in small-animal model. It was capable of quantifying metabolic turnover rates of lipoproteins in several organs simultaneously. It was also able to generate detailed 3D quantitative flow maps of the mouse brain vasculature [86]. Carbon quantum dot (CDs) have excitation dependent emission, high fluorescence quantum yields, photostability, and long photoluminescence decay lifetime. These properties make them ideal to be used in imaging modalities [87]. A versatile imaging probe with highly luminescent cadmium free *CuIn*S$e_{2}$/ZnS core/shell quantum dots conjugated to CGKRK (cys-Gly-Lys-Arg-Lys) tumour-targeting peptide was developed. It had strong tumour-specific homing property, long circulation time, excellent photostability, and minimal toxicity. It was tested on the glioblastoma mouse model and the targeted probe distinguished tumour boundaries and positively labels a population of diffusely infiltrating tumour cells. This shows their potential to be used in optical imaging [88].

#### 2.5.3. Gold Nanoparticles

The identification and characterization of disease-related mRNA in cells are of great significance for early diagnosis and treatment of numerous diseases. Oligonucleotide functionalized Au NPs have high stability, high intracellular delivery efficiency by endocytosis and high signal-to-background ratio for mRNA detection. Spherical nucleic acid Au NPs conjugates consisting of densely packed recognition oligonucleotides with complementary sequences to the target mRNA was studied. It was able to detect intracellular mRNA level [89]. Ultrasmall polyaminocarboxylate-coated Au NPs, a dithiolated derivative of diethylenetriaminepentaacetic acid, and 1,4,7,10-tetraazacyclododecan-1-glutaric acid-4,7,10-triacetic acid functionalized by thioctic acid show potential for image-guided radiotherapy. The immobilization of organic Cy5-N$H_{2}$ dyes onto the Au NPs adds radiosensitizer fluorescence properties that can be used for monitoring their internalization in cancer cells for determining their localization in cells by fluorescence microscopy. This allows for following up their accumulation in tumour after intravenous injection [90].

#### 2.5.4. Persistent Luminescence NPs

Recently, optical imaging nanoprobes are studied as contrast agents for biomedical imaging. These nanoprobes are used to provide early detection, accurate diagnosis, and treatment monitoring at the cellular and molecular level. One type of nanoprobes, called PLNPs, are developed as biomedical imaging agents (bioluminescence and fluorescence imaging), because their optical property can be varied by chemical and physical variables such as composition, size, and surface nature [2]. The PL mechanism is that when the PL materials are irradiated by light and the materials are charged until the excitation is stopped. Then, the PL materials emit light [91]. Preclinical studies on bioimaging were carried out to show that PLNPs have advantages of high signal-to-noise ratio, high sensitivity, deep penetration, and no interference from tissue autofluorescence [92,93]. For biocompatibility of PLNPs, a study was conducted on small animals using the zinc gallate (ZGO) in vivo. Mice were injected various amounts of ZGO from 1 to 8 mg. Toxicity was investigated after one day, one month, and half a year after injection. It is found that only the elevated amount of ZGO (i.e., 8 mg per mouse) would cause significant weight change in the mice, and this amount is about 5 times larger than the amount typically used for in vivo imaging [94].

### 2.6. Ultrasound

Ultrasound is a noninvasive imaging technique that can assess morphology, internal structure, orientation, and margins of the lesion from multiple planes with a high resolution both in predominantly fatty breast and dense glandular structures [95]. Ultrasound-guided drug delivery using nanobubbles (NBs) has become a promising strategy in recent years. Nanobubbles are usually composed of gas cores and stabilized shells. They can cross the capillary wall easily and have been used in many targeted therapies for cancer treatment, such as 5-fluorouracil loaded NBs for hepatocellular carcinoma. Chitosan is the N-deacetylated derivative of chitin and is one of the most abundant biological materials on earth. Chitosan NBs have gained considerable attention in cancer therapy due to their biosafety and drug transportability. A study was conducted to synthesize DOX-loaded biocompatible chitosan NBs. Nanobubbles-mediated DOX uptake and apoptosis on Michigan cancer foundation-7 cells were measured with flow cytometry and the results showed it to have excellent drug loading capability and ultrasound enhancement [96]. A new ultrasound imaging contrast agent was introduced, where the NBs were conjugated to poly (lactic-co-glycolic acid) and carried DOX as a cancer drug. The diameter of the NBs was 500 nm and the potential was −23 mV. Their multifunctionality allow it to be a great theranostic agent as well. The enhanced ultrasonic and antitumour functions were observed in in vivo results. The DOX-NBs had a drug loading efficiency of 78.6% and an encapsulation efficiency of 7.4% [97]. Bypassing the brain blood–brain barrier opening is possible with focused ultrasound. A study examined the stimulated acoustic emission of NBs at a different concentration to evaluate the blood–brain barrier opening under real-time acoustic feedback control across concentration. The study results showed that the successful opening of the blood–brain barrier was reliably achievable under real-time feedback [98]. An ultrasound-responsive phosphatidylserine-based paclitaxel liposomes NBs conjugate that has a proapoptotic effect toward enhanced anticancer efficacy and image guidance was studied. In vitro results showed the conjugate had a 10-fold increase in cellular internalization as compared to the control. The synergy between phosphatidylserine and paclitaxel (combination index, CI < 0.1) provides significantly high tumour efficacy both in vitro and in vivo (98.3 ± 0.8% tumour grown inhibition). The results also showed a significant reduction in tumour proliferation index [99]. Enhancement of macromolecular permeation through layers of retina is possible with ultrasound-responsive NBs. In one study, intracellular delivery of the antibody in the cell was quantified using Cy3-streptavidin with negligible toxicity. The results showed that macromolecular internalization was achieved to a significant amount [100]. Fluorescence upconversion NPs are highly sensitive and can function as nanocarriers. They can also label the tumour in a specific organ under NIR light. NIR has a few drawbacks to it, such as having a penetration depth of approximately 15 mm. Fluorescence in combination with ultrasound can overcome that shortcoming and provide a high-resolution signal-to-noise ratio. A system in combination with $Nd^{3+}$-sensitized upconversion NPs, graphic carbon nanodots, and NBs were used for a dual modality imaging and treatment on a mouse model [101]. Apatinib is an oral molecular antiangiogenetic medicine used to treat patients with advanced hepatocellular carcinoma. It has significant systemic toxic side effects. Ultrasound-targeted NBs destruction technology can minimize systemic drug exposure and maximize therapeutic efficacy. A study was carried out to develop novel GPC3-targeted and drug-loaded NBs for this purpose and to be used on hepatocellular carcinoma cells. The results showed ultrasound-targeted and drug-loaded NBs successfully achieved the desired destruction, selective growth inhibition, and apoptosis in HepG3 cells in vitro [102]. There was a study done to investigate the possibility of cancer therapy using a combination of NB-liposomes and ultrasound. NB-liposome was injected intratumourally, then, exposed to ultrasound (1 MHz, 0–4 W/${\mathrm{cm}}^{2}$, 2 min) in BALB/c mice that were inoculated with colon-6 cells. Tumour temperature was significantly higher when treated with NB-liposome compared to just ultrasound. It caused extensive tissue necrosis at 3–4 W/${\mathrm{cm}}^{2}$ of ultrasound exposure [103]. An oxygen encapsulated cellulosic NB agent for imaging and ultrasound-guided drug delivery was introduced. It was tested on a urothelial carcinoma model. It was propelled (max 40 mm/s) and guided oxygen NBs to the tumour using an ultrasound beam. This can localize 500 μm inside the tumour using beam guidance. It enhanced the efficacy of mitomycin-C, which yielded significantly lower tumour progression rate, while using 50% lower concentration of chemotherapeutic drug [104]. An antitumour-targeted FoxM1 siRNA-loaded cationic NBs conjugated to A10-3.2 aptamer was introduced for prostate cancer. It has high specificity to the binding of prostate-specific membrane antigen positive LNCaP cells. In vitro results showed it significantly improved transfection efficiency, cell cycle arrest, and cell apoptosis, while reducing FoxM1 expression [105].

### 2.7. Photoacoustic Imaging

Photoacoustic imaging (PAI) is based on the photoacoustic effect. It reconstructs images from captured ultrasound signals generated from the materials that thermally expand by laser pulse [106]. Photoacoustic imaging is often referred to as optoacoustic imaging. It is a low-cost modality that can provide regional imaging of blood vessels. It has high spatial and temporal resolution with clinically approved imaging depth [107].

#### 2.7.1. Gold Nanoparticles

In cancer patients, metastases rather than the primary tumour often determine tumour mortality. A new noninvasive immune functional imaging method was proposed, where ultrasound-guided PAI can be used to detect sentinel lymph node metastases with the aid of chitosan-coated Au NPs (GC-Au NPs). This was tested on tumour-bearing mice. Volumetric analysis was used to quantify GC-Au NP accumulation in the sentinel lymph node after cellular uptake and transport by immune cells. The analysis results showed that the spatial-temporal distribution of GC-Au NPs in the sentinel lymph node was affected by the presence of metastases. This imaging method can successfully detect metastatic from nonmetastatic lymph nodes using Au NPs [108]. There was a study to visualize murine lymph vessels using PAI and Au NPs as a contrast agent, and the study showed great potential for it to be used to detect sentinel lymph nodes [109]. Another study found that ultrasound-guided PAI and anti-epidermal growth factor (EGFR) antibody conjugated to gold nanorods can effectively detect EGF-expressing primary tumour and regional lymph node metastases. The nanoconjugation was tested on tumour-bearing mice. Anti-EGFR gold nanorods provided significant enhancement in PAI signal in MDA-MB-231 tumour and axillary lymph node metastases relative to MCF-7 tumour and non-lymph node metastases [110]. Moreover, a new nanorod was synthesized through seed-mediated synthesis with an aspect ratio ranging from 8.5 to 15.6. It could tune a longitudinal surface plasmon resonance absorption band that covered a broad NIR range (~680–1100nm). The gold nanorods showed good biocompatibility and stability. The nanorods provided great contrast enhancement in PAI (3.1 times to the control group) and excellent signal-to-noise ratio (5.6 times to the control group) [111]. When administering Au NPs as a contrast agent in PAI, it is important to note that the Au NPs are below the renal clearance (10 nm). A study showed that biodegradable Au NPs assembled from 5 nm primary gold particles had strong NIR absorbance. Ultra-small 5 nm Au NPs can be used to develop molecular-activated plasmonic nanosensors for molecular-specific PAI [112]. In NIR-II tissue generating the least background signal in PAI, large contrast agents in the spectral range delay their pharmacokinetics and reduce their thermal stability that yields unreliable PAI. Miniaturized Au NPs can help to solve that problem. They are 5–11 times smaller than regular gold nanorods with a similar aspect ratio in NIR-II. They are 3 times more thermally stable and can generate 3.5 times stronger PA signal under nanosecond pulsed laser illumination. These results were verified with thermotical and numerical analysis [113]. Gold nanoparticles coated with glycol chitosan (GC) can be used as a contrast agent in PAI. In breast cancer cells, GC-Au NPs have strong cellular uptake and yield a strong PA signal in a tissue phantom. After just 3 h of incubation, the phantom displayed a strong signal and did not require any additional antibodies or complex surface modification. The endocytosis of GC-Au NPs was also confirmed with dark microscopy, which is beneficial for minimizing toxicity [114]. Photoacoustic tomography (PAT) is an emerging technology that can image cells or tissue using contrast agents such as NPs and pigments. An interesting UV–vis absorption peak in NIR was observed when Au NPs were synthesized with astaxanthin. Studies showed that this astaxanthin-based Au NPs had the potential to be used in PAI and therapy [115].

In brain tumours, getting past the blood–brain barrier (BBB) is a major obstacle. The majority of contrast agents cannot get past the BBB; thus, a study was done to use gas microbubble-assisted focused ultrasound to transiently open the blood–brain barrier and locally deliver silica-coated gold nanorods across the BBB. This contrast agent had strong optical absorption, which allowed for visualization of the agent using ultrasound-guided PAI [116]. The enhancement of the amplitude of the PA signal with microbubbles conjugated to gold nanorods (Au MBs) was studied. Fluence below 5 mJ cm^−2^ provided negligible microbubble wall motion and weak PA signal. However, fluence above 5 mJ cm^−2^ produced significantly higher thermal expansion and emitted 10-fold greater amplitude PA signal compared to the control. This phenomenon can be explained by the idea that explosive boiling may occur at the nanorod surface, which produces vapor NBs and contributes to Au MBs expansion. In vivo imaging of Au MBs in a murine kidney model shows that it is an effective alternative to the existing contrast agents for PAI [117].

#### 2.7.2. Carbon Nanotube (CNT)

Carbon-based NPs have gained considerable attention due to their unique physicochemical properties in nanotechnology [118]. In one study, a single-wall CNT complex with long circulation was fabricated. It was capable of self-assembly loading of an albumin-coupled fluorescent photosensitizer and Chlorin e6 via high affinity between albumin and Evans blue, which provided them with fluorescent imaging and photodynamic ability. It was capable of providing fluorescence and PAI of tumours for optimizing therapeutic time window [119].

#### 2.7.3. Fluorescent

A metabolically digestible imaging probe was developed from nanoprecipitation of biliverdin. These NPs are composed of a biliverdin network and are cross-linked with a bifunctional amine linker. Their excitation at NIR wavelengths provides a strong photoacoustic signal. In vivo, they accumulated in a lymph node in mice and have the potential to be used as a photoacoustic agent for sentinel lymph node detection [120]. The nanocomplex consists of split fluorescent protein fragment used as a molecular glue and switchable Raman reporters to assemble Au NPs into photonic clusters. The fluorescent protein-driven assembly of metal colloids yields an enhanced PA signal that can be used as PAI agent [121]. A photoacoustic contrast agent, formulated from an FDA-approved antimycobacterial agent, clofazimine hydrochloride NPs, for a different purpose, was introduced to be used for prostate cancer. It had macrophage targeting ability and high contrast absorbance at 495 nm. The experimental results on transgenic adenocarcinoma of the mouse prostate model showed a preferential accumulation of the NPs in a cancerous prostate cell over the control. This allows PAI and analysis of prostate cancer [122]. Photoacoustic imaging has a penetration depth of a few cm and can generate useful endogenous contrast from melanin and oxy-/deoxyhemoglobin. ICG is a small molecule dye with fast clearance, bleaching effects, and rapid protein binding, and it can be used for PAI. A study was done to entrap ICG in poly (lactic-co-glycolic acid) NPs together with perfluorocarbon using a single emulsion method. The encapsulation of ICG within NPs decreases its photobleaching and increases the retention of signals within the cells. It can detect as little as 0.1 × 10^6^ cells in PAI and the nanocomplex [123]. Core–shell silica PEG NPs were developed with photothermal, photoacoustic, and NIR optical imaging properties. They were doped with triethoxysilane-derivatized cyanine 5.5 and cyanine 7 dyes, which give them photoacoustic properties. The study results showed they have outstanding stability and enhanced photoacoustic signal [124]. Photoswitchable hybrid probes with thermochromic dye and absorbing NPs were introduced where temperature-sensitive light–dark states and spectral shifts in absorption can be switched through controllable photothermal heating of doped NPs. It provided high contrast in PEI [125]. Hypoxia is often correlated with tumour aggressiveness and poor treatment outcome. Early diagnosis of hypoxic tumour cells has a high potential in tumour control. Hypoxia-activated NPs can be used to enhance the efficiency of photoacoustic intensity, fluorescence, and chemotherapy. Hypoxia-activated NPs are inactive during blood circulation and normal physiological conditions. They activate in the hypoxic condition when they extravasate into the hypoxic tumour microenvironment. Azobenzene hypoxia-activated fluorescence nanoparticles have high potential to be used in PAI [126].

## 3. Cancer Therapy

Cancer therapy is the technique of inhibition or irradiation of cancer cells. There are several techniques available and each one is more beneficial to one type of cancer treatment than others. Nanomaterials offer significant enhancement to many of the cancer therapies and they are discussed below:

### 3.1. Photothermal Therapy

Photothermal therapy is a hyperthermia-based cancer therapy. The goal of this therapy is to destroy tumour tissue while avoiding excessive heating of normal tissues. Biological tissue lacks NIR-absorbing chromophores. The use of laser wavelength in the ‘tissue optical window’ (700–1000 nm) minimizes tissue heating, while Au NPs have strong and tunable absorption in the NIR region. Therefore, Au NPs and NIR can be used to facilitate selective heating of tumours with NPs [127]. Gold nanoparticles with thiol and amine groups can be functionalized with targeted antibodies or drug products. Colloidal gold exhibits localized plasmon surface resonance. It can absorb light at specific wavelengths, which makes them useful for hyperthermic cancer treatment application. A gold nanoparticle’s localized plasmon surface resonance can be changed with the modification of the particle’s shape and size, which alters its photothermal and photoacoustic properties, allowing utilization of different wavelengths of light. Its nanosize allows the particle to localize in the tumour through passive distribution and excrete through the urinary system [128]. One of the major problems with PTT is that heat distribution is often heterogeneous throughout the tumour, which leaves part of the tumour untreated. A new idea was proposed which uses silica gold nanoshells to deliver fractionated PTT [129]. Gold-based NPs are the main mediator of PTT because they offer biocompatibility, efficient light to hear conversion, ability to be tuned to absorb NIR light which penetrates tissue more deeply, a small diameter that enables tumour penetration, and simple gold thiol bioconjugation chemistry for the attachment of the desired molecule. Nanoshells, nanocages, nanorods, and nanostars are the most common nanomaterials as photothermal transducers. The majority of Au NPs have been designed to maximally absorb within the first NIR window, which can safely penetrate 2–3 cm of tissue [130]. A PET-based nanoplatform was introduced to quantitatively correlate to the heat generation of plasmonic NPs with their potential as a cancer-killing agent. Heat generation was evaluated in human tumour xenografts in mice using 2-deoxy-2-[F-18]-fluoro-D-glucose (*^18^F—FDG*) PET imaging. The platform was validated by quantifying the photothermal efficiency of the NIR silica gold nanosphere and benchmarked it against the solid Au NPs. The results showed the heat generation of the resonant gold nanospheres (in vitro and in vivo) performed better compared to the control. It also showed PET could reliably be used to monitor early treatment response in PTT [131].

In PTT, the temperature of the tumour is raised above 42 degrees Celsius to destroy the cancer cells. A light-absorbing material or photothermal agent must be introduced into the tumour to improve the efficacy and selectivity of the energy to heat transduction. Even though gold is the most employed agent in PTT, magnetic NPs are a good alternative. Magnetic NPs formed by iron oxide can be used in combination with other substances or used by themselves as photothermal agents. They can be directed to the tumour site magnetically and their distribution in tumours and other organs can be imaged. Their molar absorption coefficient in NIR is low when they are used alone. However, this can be mitigated by clustering of the NPs. They can also be designed to release a drug upon heat generation, which can be beneficial for combination therapy of PTT and chemotherapy [132].

Polymer-based NP systems have been investigated to overcome some of the limitations associated with traditional inorganic NPs. Some of the materials that have been investigated for this purpose include polyaniline, polypyrrole, polydopamine, and poly-(3,4-ethylene dioxythiophene): poly(4-styrene sulfonate). They are often conjugated with ligands for targeting ability. A specific set of requirements should be met for NPs to be an ideal candidate for PTT, such as suitable size and uniform shape, good dispersibility in aqueous solution, respond to light in NIR range 650–950 nm to prevent damage to surrounding healthy tissue, sufficiently photostable to ensure adequate diffusion time to reach tumour before losing their photosensitivity, and exhibit low or no cytotoxicity in a living system. Current available PTT enabling agents mainly comprise metal NPs such as gold, palladium, silver, germanium, and carbon-based NPs. Some of the polymer-based NPs systems are listed in Table 1 below [133].

In PTT, red blood cell-coated NPs show improved efficacy with a faster decrease in tumour volumes and a higher survival rate than bare NPs. It is speculated that red blood cell NPs inherit the photothermal conversion effect from inner cores and the long blood retention from the red blood cell coating. One study showed that the combination of biodegradable, natural, and nontoxic melanin NPs extracted from living cuttlefish and red blood cell membrane have significantly higher PTT efficacy. Au NPs encapsulated with the antitumour drug paclitaxel-coated by anti-EpCam antibodies-modified red blood cell membranes show increased cancer-targeting ability due to anti-EpCam antibodies. Paclitaxel can be released when the membrane is destroyed by the heat generated from the Au NPs under laser irradiation to yield the anticancer effect [134].

### 3.2. Photodynamic Therapy (PDT)

Photodynamic therapy is a form of light therapy that uses molecular oxygen, visible light, and photosensitizers (PS) to destroy cancer cells and pathogenic bacteria. Photodynamic therapy is noninvasive and selectively cytotoxic to malignant cells. It causes direct tumour cell damage by apoptosis necrosis and autophagy. The photosensitizer is distributed directly into the tumour site or systematically via the vascular system. In the presence of molecular oxygen, light at a specific wavelength is applied in PDT, followed by the production of reactive oxygen species (ROS), which results in oxidative damage of the intracellular elements within the cell. This leads to cancer cell death. When PS targets the vascular system of the tumour, it results in hemostasis, vessel constriction, and breakdown. This ultimately leads to a decrease in oxygen and nutrient supply to the tumour, which eventually results in tumour cell death. Gold nanoparticles are primarily used in PDT [135]. Porphyrins have been approved for the treatment of cancer in PDT. They have low physiological solubility and lack of selectivity toward tumours, which is not efficient. Nanoparticles can be used to transport porphyrins. Silica NPs (80 nm) coated with xylan–TPPOH conjugate was studied for such purpose and showed significant phototoxic effects from post-PDT ROS generation, and stronger cellular uptake in the human colorectal cancer cell line. They showed high anticancer efficacy [136]. The dual specificity of PDT relies on the accumulation of PS in tumour tissue and localized light delivery. Tetrapyrrole structures such as bacteriochlorins, porphyrins, chlorins, and phthalocyanines with functionalization have been widely investigated in PDT. Several compounds have already received clinical approval. Photosensitizers conjugated to antibody, proteins, peptide, and other ligands with specific cellular receptors are used in targeted PDT. Nanotechnology has also been widely used for targeted delivery. Fullerene-based PS, titania photocatalysis, and the use of upconverting NPs to increase light penetration into tissue have been studied [137]. Table 2 is a list of several nanoplatforms for PDT and their advantages [138].

To achieve synergistic chemiexcited photodynamic starvation therapy against tumour metastasis, a biomimetic nanoreactor was developed. Photosensitizers on the hollow mesoporous silica NPs were excited by chemical energy in deep metastatic tumour tissue to generate singlet oxygen, and then, glucose oxidase catalyzed glucose into hydrogen peroxide in PDT. This blocked nutrient supply for starvation therapy and provided hydrogen peroxide to synergistically enhance PDT [139]. Photosensitizer chlorin e6 (Ce6) and the ferroptosis inducer erastin were self-assembled into a novel supramolecular Ce6-erastin nanodrug though bonding and π−π stacking. Ferroptosis with nanodrug enhances anticancer actions by relieving hypoxia and promoting ROS production [140].

### 3.3. Chemotherapy 

Chemotherapeutic agent DOX is a member of the anthracycline class. It is heavily used in many clinical cancer therapies. It is also one of the most used chemotherapeutic drugs for the treatment of breast cancer. Paclitaxel is another popular chemotherapeutic agent used in breast cancer. Other commonly used chemotherapy regimens are cisplatin, tamoxifen, trastuzumab, and docetaxel. The efficiency of the drug increases significantly with targeted drug delivery. Nanoparticle-based carriers are often conjugated to them for targeted delivery. Some of the NPs that are used in chemotherapy for breast cancer are polymer-based NPs, liposomal NPs, metal-based NPs (Au NPs, SPIONP), carbon-based NPs, mesoporous silica NPs, and protein-based NPs [141]. Nanoparticle vehicles are currently in clinical use and some are undergoing clinical investigation for anticancer therapies, including dendrimers, liposomes, polymeric micelles, and protein drug NPs. There are many new NPs drug formulations in development and undergoing early and late phase clinical trials, including several that utilize active targeting or triggered release based on environmental stimuli. A variety of NP formulations have been approved by the FDA and EMA for the treatment of a wide range of cancers. Some examples are pegylated liposomal doxorubicin and liposomal daunorubicin, which are available in the United States. Nonpegylated liposomal doxorubicin is approved in Europe. Nab-paclitaxel is an FDA- and EMA-approved therapy using NP albumin-bound particles [142]. Various types of proteins and small peptides are often conjugated to the surface of NPs to improve the selectivity of chemotherapeutic drugs. Serum glycoprotein is one of the targeting ligands used with NPs in chemotherapy drug delivery [143]. The antimalarial agent chloroquine can reduce the immunological clearance of NPs by resident macrophages in the liver, leading to increased tumour accumulation of the nanodrug [144].

Gold nanoparticles have high stability, surface area-to-volume ratio, surface plasmon resonance, and multifunctionalities. The nontoxic, nonimmunogenic nature, high permeability, and retention effect of Au NPs provide additional benefits by enabling penetration and accumulation of the drug at tumour sites. DOX-BLM-PEG-Au NPs and EpCAM-RPAnN are two Au NP carriers that have high potential to be used in chemotherapy [145]. Cisplatin is a genotoxic agent that can be used alone or in combination with radiation or other chemotherapeutic agents. It is used in chemotherapy for a broad range of cancers. However, the agent is limited by the intrinsic and acquired resistance, and the dose to normal tissue. It shows little selectivity for tumour vs. normal tissue, which leads to toxicity. Nanoparticles can be used to deliver cisplatin to reduce toxicity. Some organic NPs that can be used to transport cisplatin are liposomes, polymeric NPs, polymeric micelles, and dendrimers. Some inorganic NPs are Au NPs, ferromagnetic NPs, and mesoporous silica NPs. Some hybrid NPs are CNT, nanoscale coordination polymers, and polysilsesquioxane NPs [146].

Organic NPs are a popular choice for chemotherapeutic drug delivery. They can increase the circulation half-life and tumour accumulation of a drug. Combination chemotherapy is used in the treatment of a broad range of cancers. Nanoparticles are essential to delivering many of these drugs to the target site and also provide a theranostic platform for multifunction [147]. Multidrug-loaded NPs formulation consists of different classes of therapeutic agents. It has been studied for breast cancer therapy in preclinical breast cancer models. One example would be polymer lipid hybrid NPs for coencapsulated DOX and mitomycin C. It has demonstrated its efficacy in the human breast cancer model, including multidrug resistance cells. Multidrug-loaded NPs micellar formulation was also developed for the delivery of three drugs: paclitaxel, 17-AAG (Triolimus), and rapamycin. They were evaluated on MDA-MB-231 tumour-bearing mice [148]. Hypoxia promotes the invasiveness of tumour cells and chemoresistance. Tumour-associated macrophages (TAMs) reside in the hypoxic region to promote proliferation and chemoresistance. Nanoparticles Mn$O_{2}$ with high reactivity toward hydrogen peroxide for the simultaneous production of $O_{2}$ and regulation of pH can affectively alleviate tumour hypoxia by targeted delivery of $MnO_{2}$ to the hypoxic area. It was conjugated to DOX and significantly increased the apparent diffusion coefficient value of breast cancer and inhibited tumour growth [149]. A novel carrier, targeting nanomicelles for synchronous delivery of curcumin and baicalin, was introduced, which could effectively overcome tumour resistance. Mannose binds to CD206 receptors on the surface of tumour-associated macrophages, subsequently increasing the number of nanodandelions engulfed by tumour-associated macrophages. To increase tumour cellular uptake, oligomeric hyaluronic acid can also be used as a targeting material. Nanodandelions can easily enter tumour tissue through the vascular barrier due to their small size. Effective antitumour activity and reduced side effects were confirmed in antitumour experiments in A549 tumour-bearing mice [150]. Sustained-release characteristics of NPs may aid the effectiveness of chemotherapy by maintaining drug concentrations at the tumour site for longer durations. Nanoparticles can increase penetration and accumulation of the inhaled drug in tumour tissue and cells. This yield improved antitumour activity compared to the free drug. These characteristics make them suitable for chemotherapy for lung cancer [151].

### 3.4. Immunotherapy

During recent decades, cancer immune therapy has made significant progress with the improvement in nanotechnology. Immunotherapy is a therapy based on stimulation or activation of the patient’s immune system to recognize and destroy cancer cells [152]. Understanding how to increase the response rate to various classes of immunotherapy is to improving cancer treatment efficacy and minimizing adverse side effects. There are five classes of cancer immunotherapy: lymphocyte-promoting cytokines, agonistic antibodies against co-stimulatory receptors, checkpoint inhibitors, engineered T cells such as CAR T and T cell receptor (TCR) T cells, and cancer vaccines. Nanoparticles can be used to target T cells in the blood or transport mRNA to the cancer cell, or transport other vaccines in immunotherapy [153]. Nanoparticle systems have shown to be a promising tool for effective antigen delivery. The antigen is generally in peptide form that can stimulate an adaptive immune response. For conditioning a robust and long-lasting adaptive immune response, stimulation of the innate immune system through natural killer cells is necessary. Therefore, an adjuvant that works to recruit natural killer cell response is vital for effective vaccination. Table 3 summarizes the different antigens being studied for different cancer treatments and their delivery NP conjugate [154].

An adjuvant is a molecule that increases immunogenicity. They sometimes are lacking in tumour antigens when presented alone. Commonly used adjuvant in cancer treatment are 3-O-desacyk-4′-monophosphoryl lipid A (MPLA), CpG oligodeoxynucleotides (ODNs), lipopolysaccharide (LPS), polyinosinic:polycytidylic acid (poly I:C), and agonists of the stimulator of IFN genes (STING). When they are internalized in antigen-presenting cells with tumour antigens, they promote anticancer immune response [155]. Nanoparticles have a multifaceted role in modern immunotherapy. They can reduce tumour-associated macrophages and act as a tumour suppressor agent, selectively knockdown Kras oncogene addiction by the nano-Crisper-Cas9 delivery system, and serve as an efficient alternative to the chimeric antigen receptor [(CAR)-T] [156]. Immunotherapy is one of the effective modalities for cancer treatment. Targeting the tumour environment along with the immune system is a viable strategy to use for cancer treatment. Systematic delivery of immunotherapeutic agents to the body using NP delivery is of great importance. Liposomes, Au NPs, polylactic-co-glycolic acid (PLGA) NPs, micelles, iron oxide NPs, and dendrimers are widely used for immunotherapy. Polymeric NPs are the most commonly used ones in immunotherapy where PLGA is an FDA-approved polymeric carrier. Table 4 below lists the commonly used NPs used in immunotherapy, their therapeutic agent conjugate, target, function, and studied tumour model [157].

Cyclic dinucleotides (CDNs) is a potent stimulator of the interferon receptor (STING) agonist. Its efficacy is limited to micromolar concentration due to the cytosolic residence of STING in the ER membrane. Biodegradable poly (beta-amino ester) NPs were introduced to deliver CDNs to the cytosol, which leads to robust immune response > 100-fold lower extracellular CDN concentration in vitro. This NP-mediated cytosolic delivery for STING agonists synergizes with checkpoint inhibitors and has the potential for enhanced immunotherapy [158]. A new strategy of cancer immunotherapy using plant virus-based NPs was proposed. In vaccine development, plant virus has already been utilized extensively. Successful employment of plant viruses in cancer treatment has been observed using hibiscus chlorotic ringspot virus, tomato bushy stunt virus, and red clover necrotic mosaic virus. Plant viruses offer the advantage of uniformity concerning shape and size and ability to self assemble into highly repeating nanostructures. They also exhibit structurally defined chemical attachment sites, cargo capacity, and tolerance against high temperature and pH [159]. Metallic NPs also have high potential in immunotherapy. Several metallic NPs such as Au NPs have been studied to be used with several immunotherapeutic agents such as ovalbumin (OVA). Metallic NPs have also shown to improve antitumour cytotoxic T cell response. Metallic NPs have advantages which can be utilized with combination therapy of immunotherapy and PTT [160]. Elimination or reprogramming of the immune-suppressive tumour microenvironment is a major challenge in immunotherapy. Immune checkpoint inhibition targets regulatory pathways in T cells to enhance tumour response and has been the most successful method in immunotherapy. Some FDA-approved immune checkpoint agents are ipilimumab against CTLA-4, and pembrolizumab and nivolumab against PD-1. Lipid-based NPs are generally used to transport these materials to the target site [161]. A study showed that R848-loaded *β*—cyclodextrin NPs can efficiently be delivered to tumour-associated macrophages in vivo to macrophages to acquire an antitumourigenic M1-like phenotype. The functional orientation of the tumour immune microenvironment toward an M1 phenotype was achieved through the administration of CDNP-R848 in multiple mouse models. An improved immune response rate was observed when combined with immune checkpoint inhibitor anti-PD-1 [162]. Exosomes are nanosized particles secreted from most cells. This allows crosstalk between cells and their surrounding environment through cargo transfer. Tumour cells also secrete exosomes, known as tumour-derived exosomes. They have tumour modulation activity and can affect the tumour microenvironment and antitumour response. Their immunological activity influences both innate and adaptive immune systems, including regulatory T-cell maturation, natural killer cell activity, and anti-inflammatory response. Their characteristics allow them to be used for metastasis lung cancer treatment [163].

## 4. Theranostics

Theranostics involves the administration of a diagnosis agent. They are referred to as a combination of diagnosis and therapy using the same agent [164].

### 4.1. Multimodality Imaging

SPIONs, (Feraheme, FH) and [*^89^Zr*]Zr was used as a nanoplatform for PET and MRI. PET-MRI integrates the excellent sensitivity of PET with the spatial resolution and contrast of soft tissue by MRI. Feraheme can shorten the transverse relaxation time, $T_{2}$, and is generally used for dark contrast enhancement. However, dark contrast is often difficult to implement in clinical settings for applications such as detection and diagnosis of metastases in the lymph nodes. FH radiolabelled with OET tracer can take advantage of highly sensitive bright signals from PET. It can detect the presence of FH in regions, where the MRI contrast is too low or noisy. Experimental results showed that FH is a very suitable SPION for chelate-free labelling of PET tracers, and can be used in hybrid PET-MRI [165]. For combined magnetomotive ultrasound PET/CT and MRI for sentinel lymph nodes, *^68^Ga*-labelled SPIONs were proposed. The results showed that the SPIONs provided viable contrast enhancement [166]. TAM is significantly associated with poor prognosis of tumours. Using super magnetic iron oxide and perfluorocarbon nanoemulsions, quantitative monitoring of TAM is possible with MRI-based TAM imaging. A study was conducted using MRI-based measurements of TAMs as a prognostic marker and PET to observe tumour behaviour with *^18^F*-2-fluroro-2-deoxy-D-glucose as a radioactive tracer [167]. Ultra-small AGulX NPs are made of polysiloxane and are surrounded by gadolinium chelates. They are generally obtained via the top-down process. They are the first multifunctional silica-based NPs that are small enough to escape hepatic clearance. Their hydrodynamic diameter is under 5 nm, and they have excellent radiosensitizing properties for radiotherapy. They can be used in four different types of imaging modalities: MRI, SPECT, fluorescence imaging, and CT. A recent study showed that they can be used in MRI-guided radiotherapy. The study found that *^68^Ga*-AGulX@NODAGA has great potential in PET/MRI-guided radiotherapy. They can be used as a dual modality PET/MRI imaging agent with passive accumulation in the diseased area [168].

Image-guided radiotherapy can improve cancer outcomes significantly [169,170,171]. A theranostic platform and a combination of bismuth and gadolinium were proposed for onsite radiosensitization and image contrast enhancement. A study showed that NPs provided image enhancement in both CT and MRI, and tumour suppression with prolonged survival in non-small cell lung carcinoma models with minimal off-target toxicity [172]. Mesoporous silica NPs for CT and optical imaging were introduced. The high density of platinum NPs in the surface of mesoporous silica NPs greatly enhances CT contrast. NIR fluorescent dye Dy800 was conjugated to the platform to enhance optical imaging contrast. It was tested on a breast tumour mouse model. In vivo imaging showed significant enhancement in images after 24 h injection [173]. A multimodal imaging probe for PET/SPECT and MRI ($T_{2})$ was developed using SPIONs and deblock copolymer with either methoxy polyethylene glycol or primary amine $NH_{2}$ end groups. *^57^Co^2+^* ions were used as a radioactive tracer and the study results found the probe to be nontoxic [174]. Another biomedical probe with an Au NP platform was introduced that is capable of coordinating $Gd^{3+}$ for MRI and *^67^Gd^3+^* for SPECT imaging. The Au NPs had high affinity toward the gastrin-releasing peptide receptor. These receptors are overexpressed in various human cancer cells, mainly in PC3 prostate cancer cells [175]. A multifunctional targeting NP probe for pancreatic cancer was introduced that consists of 1,2 Distearoyl-sn-glycero-3-phosphoethanolamine-N-amino (polyethylene glycol)-modified SPIONs, which were conjugated with the plectin-1 antibody. In vivo optical imaging and MRI show that they highly accumulate in MIAPaCa2 and XPA-1 carcinoma cells. They can be used as a theranostic tool in fluorescence and MRI to visualize pancreatic cancer [176]. Myocardial infection (MI) is a common disease and has a high mortality rate. MnO-based NPs in conjunction with MRI and NIR fluorescence imaging can help to combat against MI. MnO possess high $r_{1}$ relaxivity and has none or minimal toxicity. They can be used as an MRI contrast agent and as a drug carrier due to their preference to accumulate in the infarcted myocardium, as shown in fluorescence imaging [177]. Dendrimers with size range between 7–12 nm have advantages over other NPs due to their improved tumour penetration ability and inclusion of a tumour-specific drug release mechanism. G5 PAMAM dendrimer can be used with a paramagnetic chemical exchange saturation transfer (PARACEST) MRI contrast agent in MRI-optical imaging as dual mode MRI-optical glioma imaging NPs. Experimental results showed they were able to identify glioma tumours at a mm scale due to the perseverance of the MRI contrast throughout the glioma [178].

Nanoparticles that have high absorption in the NIR region are valuable in biomedical applications. Photoacoustic imaging (PAI) is an imaging modality that makes use of optical excitation. The imaging provides deep tissue penetration and high spatial resolution. In PAI, the photoacoustic signal is primarily determined by the pulsed laser. Therefore, the contrast agents used in PAI generally can also be used in PTT. Mesoporous carbon nanospheres (Meso-CNS), as a stable suspension with broadband and intense absorption in the UV–vis–NIR region, were studied. The analysis of photothermal conversion and photoacoustic generation show Meso-CNS possess absorption coefficients that are 1.5–2 times higher than those of CNT and graphene in the broad wavelength region, and comparable to gold nanorod in both NIR-I and NIR-II region. They can efficiently (35 wt%) load DOX due to their large surface area, appropriate pore volume, and size. All of these characteristics make them an excellent theranostic platform [179]. A dual-mode imaging system of photoacoustic microscopy and fluorescence optical microscopy with Au NPs was also proposed. Gold nanoparticles have a large absorption coefficient and enough fluoresce emission with a wavelength of 512 nm. They can be used to label certain drugs in tobacco cells, and also can be used to carry the labelled drug in the target position [180].

### 4.2. Image-Guided Therapy

A semiconducting plasmonic nanovesicle was proposed that consisted of semiconducting poly (perylene diimide) (PPDI) and poly(ethylene glycol (PEG) tethered to Au NPs (Au@PPDI/PEG). The complex was highly localized and had a strong electromagnetic field between adjacent Au NPs in the vesicular shell. The electromagnetic field enhanced the light absorption efficiency of PPDI. It generates a great photothermal effect. It also provides a strong photoacoustic signal that can be used in PAI. Overall, the complex has high potential as a theranostic agent [181]. Gold nanorods in PAI and plasmonic PTT have been studied. The advantageous properties of Au NPs such as biocompatibility, tuneable surface plasmonic resonance, and controlled synthesis make them a great choice for theranostic applications. PAI-guided PTT is possible when the pulse is used to destroy the cancer cells. This application has great potential to be used for lung cancer [182]. A hybrid reduced graphene oxide (rGO)-loaded ultra-small plasmonic gold nanorod vesicle (rGO-AuNRVe) had excellent photoacoustic signal amplification ability, and the photothermal effect was proposed as a theranostic tool to be used in PIA, PTT, and chemotherapy. It had high DOX loading capability and efficiency, and it can unload upon light NIR photothermal heating. This ability makes them ideal for a combination of photochemotherapy. When rGO-AuNRVe was labelled with *^64^Cu*, it showed high accumulation in U87MG tumours via passive accumulation in PET imaging [183]. Pure bismuth NPs have ultrahigh x-ray attenuation coefficient and light to heat conversion capabilities. These characteristics make them suitable to be used in PAI and PTT. In one study, bismuth NPs were able to increase the temperature by 70 degrees Celsius within 4 min under infrared irradiation in PTT [184]. Carbon nanotubes have advantageous optical, thermal, mechanical, electrical, and magnetic properties. Some of the applications of CNT in biology are as a heating agent, contrast agent, and drug delivery agent. Carbon nanotubes can increase the temperature in the tissue during laser irradiation in PTT, and at the same time, enhance photoacoustic signals. The nanotubes can potentially be used as a theranostic agent in PTT and PAI [185]. A theranostic agent was developed that consists of perfluorohexane liquid and Au NPs that make up the core and is stabilized by a polymer shell (poly (lactide-co-glycolic acid)(PLGA)). When PLGA-Au NPs localize in tumour cells and are exposed to laser pulses, cell viability decreases, leading to cell death. The study results showed they have viable potential to be used as a PAI and therapeutic agent for future clinical cancer therapy [186].

Gold nanoparticles have a high atomic number and they strongly absorb low and medium energy x-rays by the photoelectric effect [187,188,189]. During the photoelectric effect, characteristic x-rays and Auger electrons released in the surroundings are in a short-range. They can cause additional local damage [190]. Gold nanoparticles can be conjugated to targeting ligands or they can selectively be accumulated into the tumour via passive permeability and retention effects. Due to these abilities, they have high potential to be very effective in tumour radiotherapy augmentation without increasing the dose to the surrounding normal tissues, and can also be used as a contrast agent in CT, making it an excellent theranostic tool [191,192]. A hyaluronic acid-functionalized bismuth oxide NP was synthesized using a one-pot hydrothermal method used in targeting specific CT imaging and radiosensitizing of tumour. The integration of hyaluronic acid $Bi_{2}O_{3}$ NPs provides solubility in water and excellent biocompatibility. Targeting mechanism allows them to be taken up specifically by CD44 receptors overexpressed in cancer cells. HA-$Bi_{2}O_{3}$ NPs have high x-ray attenuation efficiency. They also have ideal radiosensitivity through synergizing x-rays to induce cell apoptosis and arrest cell cycle in a dose-dependent manner. A study showed that these active targeting NPs provide excellent CT imaging enhancement and can be used as a theranostic tool [193].

A liposome-coencapsulated DOX, hollow Au NPs, and perfluorocarbon were synthesized into a theranostic agent. It had an efficient light-to-heat conversion effect under 808 nm NIR laser irradiation and small size that enabled high accumulation in the tumour sites. It also had an efficient DOX release and enhanced ultrasound signal. All of these properties make it an excellent theranostic agent in PTT and ultrasound imaging [194]. Another ultrasonic photothermal agent was introduced that consists of NBs, graphene oxide, and hairpin sulfate proteoglycan glypican-3 (target molecule). It can work as a molecularly targeted contrast agent and contrast enhancer for ultrasound imaging. It can also be used in combination with ultrasound and PTT [195]. A new NBs–paclitaxel liposome complex for ultrasound imaging and ultrasound responsive drug delivery was introduced. The complex was 528.7 ± 31.7 nm in size with paclitaxel entrapment efficiency of 85.4 ± 4.39%, and conjugation efficiency of ~98.7 ± 0.14% with 200 nm-sized liposomes. When treated with the NBs–paclitaxel liposome, the sonoporation of MiaPaCa-2 cells had 2.5-fold higher uptake of liposomes compared to the control. It also had more than 300-fold higher anticancer activity compared to the commercial formulation ABRAZANE. The conjugate exhibits echogenicity comparable to the commercial ultrasound contrast agent SonoVue, where the echogenic stability of NBs–paclitaxel was more than one week. These properties and the image enhancing properties make it an excellent theranostic agent [196].

Silica NPs have been intensively studied in drug delivery and can be integrated with other materials for theranostic capabilities. The MnO/$SiO_{2}$ core–shell can be used for multimodal imaging. Its localization can be monitored with MRI and poly (propylene fumarate) scaffolds. The anticancer drug DOX can be loaded into it. Its porous silica shell enhances the water dispersibility of the core and minimizes leakage of the core iron [197]. Carbon dots are widely used in optical imaging nanoprobes. They are generally used for labelling cells in cancer treatment. A study proposed a gadolinium complex that consists of carbon nanodots. They have high fluorescent properties, excellent water solubility, and biocompatibility. They can also be conjugated to apoferritin nanocages for drug loading capabilities such as DOX. Folic acid can be used as a targeting molecule for MCF-7 cells and the results showed it is a viable theranostic tool with negligible toxicity [198]. A new iron (III)–tannic complex-based NP (Fe–TA NP) was introduced. It had good physicochemical properties with the capability of inducing autophagy in both hepatocellular carcinoma cells (HePG2.2.15) and normal rat hepatocytes (AML12). Experimental results showed the Fe–TA NP was capable of inducing HepG2.2.15 cell death via autography and did not affect cell viability in AML12 cells due to much higher uptake of the Fe-TA NPs by the HepG2.2.15 cells. Enhancement of the $T_{1}$ MRI contrast was achieved in HepG2.2.15 cells due to these circumstances. These results also suggest that the Fe–TA NP can provide new strategies for combining diagnostic and therapeutic functions for hepatocellular carcinoma [199].

A synergistic platform for synergistic therapy and real-time imaging was studied. It is very advantageous when treating cancer patients. However, it also faces many challenges for clinical use. Novel theranostic agent, bismuth sulfide@mesoporous silica ($Bi_{2}S_{3}@mPs)$ core–shell NPs were introduced to be used in targeted image-guided therapy for EGFR-2 positive breast cancer. The agent was obtained by decorating polyvinylpyrrolidone with $Bi_{2}S_{3}$ NR. It was chemically encapsulated with a mesoporous silica layer loaded with DOX, an anticancer drug. Trastuzumab was used as a targeting molecule that targets EGFR-2. They overexpressed in breast cancer cells. Experimental results showed the agent has good drug loading capabilities, biocompatibility, strong x-ray attenuation of the bismuth element, and precise tumour targeting and accumulation. These characteristics allow it to simultaneously act as a contrast enhancer for CT in deep tissue and as a therapeutic agent in synergistic photothermal chemotherapy [200]. Another synergistic treatment platform was developed for PAI, targeted PTT, and chemotherapy. Its use was studied in triple-negative breast cancer. The nanoplatform was composed of magnetic hybrid NP (lipid, doxorubicin), gold nanorods, and an iron oxide nanocluster (LDGI) loaded with mesenchymal stem cells. LDGIs have efficient cellular uptake by stem cells and are still be able to maintain their cellular function. LDGI can simultaneously release drugs and achieve photothermal properties upon light irradiation. The drug can then enter the cell and activate cell apoptosis. Mesenchymal stem cells have the highest enhanced migration and penetration abilities in tumours. It also showed the best antitumour efficacy in chemophotothermal therapy compared to other treatment groups in triple-negative breast cancer [201]. Another study showed a new strategy to use gold nanorod conjugated with polyacrylic acid/calcium phosphate (AuNR@PAA/CaP) yolk-shell NPs for dual-mode x-ray CT/PAI and PTT. It possesses extremely high DOX loading capabilities, pH and NIR dual responsive drug delivery ability, and high photothermal conversion properties. At low pH, the CaP shell takes damage and releases DOX. When the conjugate is exposed to NIR irradiation, burst-like drug release occurs [202]. A human cytokine-induced killer cell (CIK) was loaded with gold nanorods that were used for targeted PAI, enhanced immunotherapy, and PTT for gastric cancer. The study results showed that CIK-labelled gold nanorods actively target gastric cancer MGC803 cells and activate cell apoptosis under NIR laser irradiation. The results also showed CIKAuNR can actively target and image subcutaneous gastric cancer vessels via PIA after 4 h of injection. It can also enhance immunotherapy by regulating cytokines and kill gastric cancer cells by PTT [203]. SPIONs have high $r_{1}$and $r_{2}$ relativities and they can be completely eliminated from the body. They can accumulate in cancer through passive targeting permeability, and retention effects or active targeting. The magnetite and maghemite cores of SPIONs can easily be detected with MRI. Polymer coating SPIONs can be loaded with therapeutic agents to facilitate MRI-guided drug delivery, PTT, PDT, gene therapy, or magnetic hyperthermia. SPIONs-delivered chemotherapy has high potential, and a variety of small chemotherapeutic agents have been incorporated into SPIONs-based nanocarriers through a cleavable linker or π–π stacking. They can increase blood circulation half-life, promote tumour retention, and enable real-time drug tracking. Accumulation of SPIONs in the spleen or other reticuloendothelial systems can exert toxic effects after multiple-dose administration. Smart or responsive SPIONs have been developed to mediate that problem. SPIONs can also be used as a carrier for small interfering RNA (siRNA) or microRNA (miRNA), which can protect the ribonucleic acid and prevent enzyme degradation [204].

### 4.3. Combination Therapy

Combination therapy provides treatment of several malignancies to improve clinical outcomes. They generally induce synergistic drug action and try to work around drug resistance. There are several NPs used in combination therapy such as liposomes. Various liposomal formulation of DOX include DaunoXome, Doxil, DepoCyt, and ONCO-TCS. Liposomes are one of the most established drug delivery vehicles, with many clinical products. Some other NPs that are used in combination therapy are polymeric NPs. They have high thermodynamic and kinetic properties used in site-specific delivery of the anticancer drug to tumours. Metallic NPs, dendrimers, nanodiamonds, carbon NPs, and CNT are some other ones used in combination therapy [205]. A mesoporous NP-based drug delivery system was introduced to be used for real-time imaging in photothermal/photodynamic therapy and nanozyme oxidative therapy. In one study on synthesized mesoporous carbon–gold hybrid nanozyme nanoprobes, carbon nanospheres were doped with small Au NPs, and stabilized with a complex of reduced serum albumin and folic acid. They were then loaded with IR780 iodide. Their large surface area and numerous -COOH groups allowed for chemical modification for numerous targeting molecules, load abundant NIR dye, and photothermal agents. Small Au NPs were utilized as nanozymes to catalyze $H_{2}O_{2}$ located in the tumour cells to generate OH for intracellular oxidative damage to the tumour. In vivo and vitro results showed the nanoprobe had excellent tumour targeting efficacy, long tumour retention, and favorable therapeutic effect [206]. For combined PDT/PTT with photodecomposable, photothermal, and photodynamic properties, $SP^{3}NP$s were prepared from self-assembled PEGylated cypate that consists of PEG and ICG derivate. It can generate singlet oxygen for PDT and photothermal effect for PTT. It has high accumulation in tumour due to PEGylated surface and small size (~60 nm). All of these properties make it a potential candidate to be used in image-guided PDT/PTT [207].

Chemotherapy is one of the most common cancer treatment options, but it has showed off-target toxicity issues. Theranostic NPs integrates diagnostic and therapeutic functions within one platform, increases tumour selectivity for more effective therapy, and assists in diagnosis and monitoring of therapeutic response. Core–shell NPs were synthesized by nanoprecipitation of blends of the biodegradable and biocompatible amphiphilic copolymers poly (lactic-co-glycolic acid)-b-ply-L-lysine and poly (lactic acid)-b-ply (ethylene glycol). The NPs were spherical and had an average size of 60–90 nm. DOX was encapsulated in the core of the NPs. The results showed a 33-fold increase in NIR fluorescence in the mouse model and found it to be suitable for a controlled drug delivery system and a contrast agent for imaging cancer cells [208]. PTT in combination with chemotherapy can trigger powerful antitumour immunity against tumours. Polydopamine-coated spiky Au NPs with high photothermal stability were introduced for PTT and chemotherapy. A single round of PTT combined with a subtherapeutic dose of DOX can yield good antitumour immune response and eliminate primary and untreated distant metastasis in 85% of animals bearing CT26 colon carcinoma. Their efficacy was studied against TC-1 submucosa-lung metastasis, a highly aggressive model for advanced head-and-neck squamous cell carcinoma [209]. Monotherapy of cancer is usually subjected to some sacrifice and as such, limits therapeutic benefits. Generally, in the form of systemic toxicity, a combination of chemo and PTT elevates the therapeutic benefits and is generally considered a maximal cooperation effect achieved in combination therapy. Silica NPs with Cetuximab to target the epidermal growth factor receptor were developed. They had a high drug loading capacity of Cet-SLN that can be used to encapsulate photothermal agent ICG. It can simultaneously codeliver ICG and Cet for combinational chemophotothermal therapy of breast cancer [210]. Approximately 90% of the cancer therapeutic failure in patients is due to chemoresistance. Some cancer cells such as progenitor cells or cancer stem cells develop radioresistance from a variety of chemotherapy agents. Chemo agents generally aim to destroy rapidly dividing cells and do not have much effect on undifferentiated cancer stem cells. Hepatocellular carcinoma is responsible for the third leading cause of cancer-related death and the fifth most common type of cancer. Gold nanorods have been studied to provide a solution. Gold nanoparticles in conjunction with PTT can destroy these cells and gold nanorods can provide suitable contrast agents for PAI. Gold nanoparticles can also act as a carrier. They can carry therapeutic agents such as Adr when in conjunction with EpCAM antibody on the surface of the nanosystem. Adr/AuNPs@Pms-antiEpCAM can specifically target cancer stem cells and enhance the concentration of drugs in the tumour. This complex can be useful as a future theranostic tool [211]. Modification of NPs allows administration of the drug across the brain and provide a theranostic platform for Alzheimer’s, Parkinson’s, Huntington’s, and epilepsy disease. NPs can be used as a carrier to get past the blood–brain barrier and deliver the drug to the brain [212].

Photoacoustic and fluorescence imaging in NIR-II hold great potential due to their noninvasive nature and excellent spatial resolution properties. NIR-II is superior in biological imaging due to its higher signal-to-noise ratio and deeper tissue penetration. Photoacoustic imaging in NIR-II allows direct and wide visualization of dynamic biological tissues with high spatiotemporal resolution and sensitivity. It cannot provide comprehensive and accurate diagnosis information, so fluoroscopic imaging in NIR-II can make up the missing information in this dual imaging system. It can be used to facilitate image-guided synergistic chemophotodynamic therapy using gold nanorods. It can also be used as a carrier and allow precise controlled *^1^O_2_* drug release [213]. Nanoscale coordination polymer core–shell NPs carry oxaliplatin in the core and photosensitize the pyropheophorbide–lipid conjugate in the shell for effective chemotherapy and PDT. The synergy between oxaliplatin and pyrolipid-induced PDT kills tumour cells and provokes an immune response. This results in calreticulin exposure on the cell surface, antitumour vaccination, and an abscopal effect [214].

Photothermal therapy can be an effective antitumour therapy but it may not eliminate tumour cells. This can lead to the risk of recurrence or metastasis. Photothermal therapy in combination with immunotherapy can minimize that risk. Polydopamine-coated $AI_{2}O_{3}$ NPs were introduced for this type of combination therapy. NIR laser irradiation can kill the majority of the tumour tissue via PTT. It also releases tumour-associated antigens. The $AI_{2}O_{3}$ within the NPs, together with CpG that acts as an adjuvant to trigger robust cell-mediated immune responses, can help eliminate the residual tumour cells. Fifty percent of mice, after going through combined therapy, achieved goal tumour eradication and survived for 120 days, which was the end goal of the experiment [215]. Photothermal therapy can be combined with blockage checkpoints to achieve an even more enhanced antitumour effect. Table 5 below summarizes different NPs that can be used in photothermal immunotherapy [216].

ICG is a photothermal agent and imiquimod (R837) is a toll-like receptor-7 agonist. In one study, they were coencapsulated by poly (lactic-co-glycolic) acid (PLGA). The formed NPs were composed purely by three clinically approved components that can be used for NIR laser-triggered photothermal elimination of primary tumours. This generates tumour-associated antigens, which in the presence of adjuvant R837-containing NPs, show vaccine-like functions. In combination with the checkpoint blockade using anticytotoxic T-lymphocyte antigen-4 (CTTLA4), the generated immunological responses will be able to eliminate remaining tumour cells and will be very useful in metastasis inhibition [217]. Metastatic breast cancer is one of the most devastating cancers and has very limited therapeutic options. Nanoparticle-based platforms can offer some therapeutic options for it with different combinations of therapy. The chemotherapeutic drug DOX can be delivered using NPs. PTX formulated with albumin to form NPs is currently used in the clinic for breast cancer therapy. siRNA can also be delivered using NPs for gene therapy. Nanoparticles offer an option for photothermal therapy and magnetothermal therapy. They can also be used as a contrast enhancement agent for image-guided radiotherapy [218]. Gastric cancer is the second most malignant tumour in the world. HER-2 is one of the key targets for gastric cancer therapy. A gold nanoshell drug carrier was developed for delivery of immunotherapeutic agent and selective photothermal release of genes that targets HER-2 and the immunologic adjuvant CPG sequence in gastric tumour cells. This allows multidimensional treatment strategies such as gene, immune, and PTT. The study results showed good gene transduction ability and combined treatment effect [219]. A nanosystem consisting of ER targeting pardaxin peptides modified ICG conjugated to hollow gold nanospheres, together with oxygen delivering hemoglobin liposome was studied in PDT, PTT, and immunotherapy. It induces robust ER stress and calreticulin exposure on the cell surface under NIR light irradiation. CRT, a marker for ICD, acts as an eat-me signal to stimulate the antigen-presenting function of dendritic cells. It triggers a series of immunological responses including cytotoxic cytokine secretion and CD$8^{+}$ T cell proliferation [220]. A theranostic nanoplatform that was capable of PAI, as well as a combination of gene and photothermal therapy, were studied. A gold nanorod was coated with dipicolyl amine, which forms stable complexes with $Zn^{2+}$ cations and yields a Zn (II) dipicolyl amine gold nanorod. It has a strong complexation with anti-polo like kinase 1 siRNA used for gene silencing. The Au NPs can act as a photothermal agent as well as an enhancer for photoacoustic imaging upon laser irradiation. Experimental results showed that they yield significant antitumour activity in the PC-3 tumour mouse model [221].

## 5. Conclusions

Recent advances in nanotechnology have resulted in great progress of synthetic techniques, which benefit from the design of many nanomaterials, such as nanoparticles, nanocages, nanodiamonds, nanoshells, and nanotubes. These nanomaterials can act as very effective contrast agents in various medical imaging modalities and provide a large number of options in modern cancer therapy. The nanomaterials allow delivery of many drugs to target sites that otherwise would not be possible and provide a fundamental basis for some cancer therapy that is showing promising clinical outcomes. It is expected that continuous discovery in nanotechnology will significantly influence future cancer therapy and medical imaging. However, some of the limitations of nanomaterials as drug carriers, contrast agents, and sensitizers, such as cytotoxicity and nonbiodegradability, should be studied further in order to minimize the side effects on humans.

For the transition of nanomaterial applications in biomedical imaging and cancer therapy into commercial clinical practice, it can be seen that many in vitro and in vivo studies have shown promising results. However, numerous challenges, such as physicochemical properties, drug metabolism, cytotoxicity and biocompatibility, pharmacokinetic screening, surface engineering, in vivo efficacy, nanomaterial uptake, immunogenic issues, and preparation costs, still remain. The mechanisms of action such as the potential impact on the cellular communication, which would limit its clinical transformation, are still unclear. Based on the above challenges, possible future directions include further optimizing various nanomaterials and elucidating the precise mechanisms between the cell and nanomaterials, to achieve better imaging and therapeutic effects, and accelerate the translation of nanomaterials into clinical practice.

## Figures and Tables

**Table 1:** Polymer-based nanoparticle system for PTT [133].

Polymer	Configuration	Testing Stage
Polyaniline	NPs	In vitro and in vivo
	F-127 Conjugated NPs	
	Silver core, Polyaniline shell (ICG-Ag@PANI)	
	NPs with lanreotide and methotrexate (LT-MTX/PANI NPs)	
	WS core, polyaniline shell with hyaluronic acid and chlorin e6 (Ce6)	
	Polyaniline and cisplastin within folate-poly (ethylene glycol)-distearoylphosphatidylcholine (FA-PEG-DSPE), cRGD[cyclic (Arg-GLY-Asp-D-Phe-Lys)]-PEG-DSPE, and lecithin conjugates dubbed FA/cRGD-PNPs	
Polydopamine	Dopamine-melanin colloidal nanospheres	In vitro and in vivo
	PEGylated polydopamine NPs conjugated with ICG (PDA-ICG-PEF) loaded with DOX	In vitro
	Pegylated NPs loaded with 7-ethyl-10-hydroxycamptjotheticin (SN38)	In vivo
	DOX encapsulated with DSPE-PEG micelles coated with polydopamine	In vitro and in vivo
	Fe(3)O(4) core polydopamine coated nanoshell	In vitro
	Polydopamine coated gold nanorods	In vitro
	Polydopamine coated gold/silver NPs	In vitro
Polypyrrole	Base NPs	In vitro and in vivo
	Base NPs	In vitro
	Spindle-like hollow polypyrrole nanocapsules (PPy HNCs) loaded with DOX	In vivo
	Ppy and rapamycin loaded into liposomes conjugated with trastuzumab (LRPmAB)	In vitro
TBDOPV-DT	D-A conjugated polymer (TBDOPV-DT), with 2,2-bithiophene serving as a donor and thiophene-fused benzo-difuran dione-based oligo (p-phenylenevinylene) as an acceptor (TBDOPV-DT NPs)	In vitro and in vivo
PEDOT:PSS	PEGylated PEDOT:PSS NPs (PDOT:PSS-PEG)	In vivo
	PEDOT: PSS-PEG loaded with DOX, SN38, and Ce6	In vitro
	Magnetic NPs with PEDOT: PSS Cyanine7 (Cy7), and 2-deoxyglucose (2-DG)-polyethylene glcol (MNP@PES-Cy7/2-DG)	In vitro and in vivo
	Magnetic NPs with PEDOT: PSS coating	In vivo

**Table 2:** Nanoplatforms for PDT and their advantages [138].

Nanoparticle Platform	Advantages
Passive PDT PS tumour drug micelles and Liposomes	Enhanced tumour uptake and improved phototoxicity
Dendrimer encapsulated NPs	High loading drug
Metal oxide NPs	High loading capability, biocompatibility, easy surface modification
Immuno NPs	The highly specific molecule, improved drug release within the desired cell
Quantum dots	Large absorbance cross-section and size-tunable optical properties

**Table 3:** Nanoparticle vaccine delivery for various cancer [154].

Cancer Type	Nanoparticles	Antigen
Melanoma	Poly(lactic-co-glycolic acid) (PLGA) NPs	Ag, Poly(I:C)
	Liposome	TRP2, α GalCer
	CNT	α CD40, CpG
	Cowpea mosaic virus (CPMV) NPs	Empty Cowpea mosaic virus (eCPMV)
Non-small cell lung cancer	L-BLP25 liposome	MUC1
Breast cancer	PLGA-PEG	Ovalbumin (OVA), Monophosphoryl lipid A (MPLA), CpG
Prostate cancer	Virus-like particle	Polyethylenimine-stearic acid (PSA)
Cervical cancer	Tumour virus vaccine	HPV

**Table 4:** Nanoparticle used in immunotherapy [157].

NP Materials	Therapeutic Agents	Target	Function	Tumour Model
PLGA-based NPs	AUNP12 anti-PD-1 peptide	Tumour cells	Blockage of PD-1/PDL-1 pathway	4T1 Subcutaneous tumour
	Trastuzumab	Human epidermal growth factor 2 (HER2)	GER2 degradation and antibody-dependent cell-mediated cytotoxicity	In vitro HER2 Positive breast model
	Pam3CSK4 and α-CD40-mAb	CD40	T cell response	B16-OVA Subcutaneous tumour
Liposomes	SB505124 TGF-*β* 1 inhibitor	Tumour specific cytotoxic T-lymphocyte CTLs	Block TGF-*β* Signal and promote CD8 + T cell infiltration	E.g7-OVA Subcutaneous tumour
	Curdlan and mannan	Cytosol of DCs	Activation of DCs via Th1 cytokine production	DC2.4 in vitro model
	Stimulator of interferon genes (STING) agonists and c GAMP	Tumour microenvironment (TME)	Proinflammatory gene induction and production of immunological memory	B16-F10 Lung metastatic tumour
Micelles	Pyranine antigen	Cytoplasm of DCs	Antigen-specific cellular immunity	C57BL/6 intradermal immunized mice
	NLG919/IR780	Lymph node	Suppression of growth of tumour margin in primary tumours	4T1 subcutaneous tumour
	ROS inducing ZnPP PM/PIC	Tumour-associated macrophages (TAMs)	Activation of NK cells and T lymphocytes	B16-F10 Subcutaneous tumour
AuNPs	OVA peptide antigen/CpG adjuvant	Dendritic cells	Induce systemic antigen-specific immune response	B-16 OVA Subcutaneous tumour
	α-PDL1	Tumour cells	Imaging and tumour reduction	Colon cancer Subcutaneous tumour
Iron Oxide NPs	Superparamagnetic $Fe_{3}O_{4}$	DCs and macrophages	Immune cell activation and cytokine production	CT2 Subcutaneous tumour
	Ferumoxytol	Macrophages	Increased caspase-3 activity and proinflammatory Th1 response	MMTV-PyMT Mammary tumour
Dendrimers	mAbK1/PTX	Tumour cells—mesothelin receptor	Specific binding and antitumour activity	OVCAR3 Subcutaneous tumour
Artificial exosomes	DEC205 monoclonal antibody	Dendritic cells	Targeting to DCs	In vitro studies-DCs

**Table 5:** Nanoparticles used in photothermal therapy (PTT) immunotherapy [216].

Photothermal NPs	Checkpoint Blockade	Effector Cells	Tumours
Prussian blue NPs	Anti-CTLA-4	CD$4^{+}$/CD$8^{+}$ T cells	Neuroblastoma
PEGylated single-walled nanotubes	Anti-CTLA-4	$\mathrm{DCs},\;\mathrm{CD}4^{+}$$/\mathrm{CD}8^{+}$$\;T\;\mathrm{cells},\;\mathrm{CD}20^{+}\;$TILs	4T1 murine breast tumour, murine B16 musculus skin melanoma
PLGA-ICG-R837 NPs	Anti-CTLA-4	DCs, CD$4^{+}$/CD$8^{+}$, memory T cells	4T1 murine breast tumour, CT26 colorectal cancer
Gold nanostars	Anti-PD-L1	$\mathrm{CD}4^{+}$$/\mathrm{CD}8^{+}$$\;T\;\mathrm{cells},\;\mathrm{CD}19^{+}$ B cells	MB49 bladder tumour

## References

[1] Rosado-De-Castro P.H., Morales M.D.P., Pimentel-Coelho P.M., Mendez-Otero R., Herranz F. (2018). Development and application of nanoparticles in biomedical imaging. Contrast Media Mol. Imaging.

[2] Lecuyer T., Teston E., Ramirez-Garcia G., Maldiney T., Viana B., Seguin J., Mignet N., Scherman D., Richard C. (2016). Chemically engineered persistent luminescence nanoprobes for bioimaging. Theranostics.

[3] Liu J., Lécuyer T., Seguin J., Mignet N., Scherman D., Viana B., Richard C. (2019). Imaging and therapeutic applications of persistent luminescence nanomaterials. Adv. Drug Deliv. Rev..

[4] Baetke S.C., Lammers T., Kiessling F. (2015). Applications of nanoparticles for diagnosis and therapy of cancer. Br. J. Radiol..

[5] Kim J., Lee N., Hyeon T. (2017). Recent development of nanoparticles for molecular imaging. Philos. Trans. R. Soc. A Math. Phys. Eng. Sci..

[6] Burke B.P., Cawthorne C., Archibald S.J. (2017). Multimodal nanoparticle imaging agents: Design and applications. Philos. Trans. R. Soc. A Math. Phys. Eng. Sci..

[7] Yousaf T., Dervenoulas G., Politis M. (2018). Advances in MRI Methodology. Int. Rev. Neurobiol..

[8] Hemond C.C., Bakshi R. (2018). Magnetic resonance imaging in multiple sclerosis. Cold Spring Harb. Perspect. Med..

[9] Behzadi A.H., Farooq Z., Newhouse J.H., Prince M.R. (2018). MRI and CT contrast media extravasation. Medcine.

[10] Esqueda A.C., López J.A., Andreu-de-Riquer G., Alvarado-Monzón J.C., Ratnakar J., Lubag A.J., Sherry A.D., De León-Rodríguez L.M. (2009). A new gadolinium-based MRI zinc sensor. J. Am. Chem. Soc..

[11] Lux J., Sherry A.D. (2018). Advances in gadolinium-based MRI contrast agent designs for monitoring biological processes in vivo. Curr. Opin. Chem. Biol..

[12] Liu X., Madhankumar A.B., Miller P.A., Duck K.A., Hafenstein S., Rizk E., Slagle-Webb B., Sheehan J.M., Connor J.R., Yang Q.X. (2016). MRI contrast agent for targeting glioma: Interleukin-13 labeled liposome encapsulating gadolinium-DTPA. Neuro. Oncol..

[13] McMahon M.T., Bulte J.W.M. (2018). Two decades of dendrimers as versatile MRI agents: A tale with and without metals. Wiley Interdiscip. Rev. Nanomed. Nanobiotechnol..

[14] Moghimi H., Zohdiaghdam R., Riahialam N., Behrouzkia Z. (2019). The assessment of toxicity characteristics of cellular uptake of paramagnetic nanoparticles as a new magnetic resonance imaging contrast agent. Iran. J. Pharm. Res..

[15] Rogosnitzky M., Branch S. (2016). Gadolinium-based contrast agent toxicity: A review of known and proposed mechanisms. BioMetals.

[16] Layne K.A., Dargan P.I., Archer J.R.H., Wood D.M. (2018). Gadolinium deposition and the potential for toxicological sequelae – A literature review of issues surrounding gadolinium-based contrast agents. Br. J. Clin. Pharmacol..

[17] Ramalho J., Semelka R.C., Ramalho M., Nunes R.H., AlObaidy M., Castillo M. (2016). Gadolinium-based contrast agent accumulation and toxicity: An update. Am. J. Neuroradiol..

[18] Wei H., Bruns O.T., Kaul M.G., Hansen E.C., Barch M., Wiśniowska A., Chen O., Chen Y., Li N., Okada S. (2017). Exceedingly small iron oxide nanoparticles as positive MRI contrast agents. Proc. Natl. Acad. Sci. USA.

[19] Dulińska-Litewka J., Łazarczyk A., Hałubiec P., Szafrański O., Karnas K., Karewicz A. (2019). Superparamagnetic iron oxide nanoparticles-current and prospective medical applications. Materials.

[20] Kim S.J., Lewis B., Steiner M.S., Bissa U.V., Dose C., Frank J.A. (2016). Superparamagnetic iron oxide nanoparticles for direct labelling of stem cells and in vivo MRI tracking. Contrast Media Mol. Imaging.

[21] Iyer S.R., Xu S., Stains J.P., Bennett C.H., Lovering R.M. (2017). Superparamagnetic iron oxide nanoparticles in musculoskeletal biology. Tissue Eng. Part. B Rev..

[22] Kumar S., Kumar B.S.H., Khushu S. (2016). Increased transverse relaxivity in ultrasmall superparamagnetic iron oxide nanoparticles used as MRI contrast agent for biomedical imaging. Contrast Media Mol. Imaging.

[23] Hobson N.J., Weng X., Siow B., Veiga C., Ashford M., Thanh N.T.K., Schätzlein A.G., Uchegbu I.F. (2019). Clustering superparamagnetic iron oxide nanoparticles produces organ-Targeted high-contrast magnetic resonance images. Nanomedicine.

[24] Shari S., Seyednejad H., Laurent S., Atyabi F. (2015). Superparamagnetic iron oxide nanoparticles for in vivo molecular and cellular imaging. Contrast Media Mol. Imaging.

[25] Wang Z.J., Ohliger M.A., Larson P.E.Z., Gordon J.W., Bok R.A., Slater J., Villanueva-Meyer J.E., Hess C.P., Kurhanewicz J., Vigneron D.B. (2019). Hyperpolarized 13C MRI: State of the art and future directions. Radiology.

[26] Kurhanewicz J., Vigneron D.B., Ardenkjaer-Larsen J.H., Bankson J.A., Brindle K., Cunningham C.H., Gallagher F.A., Keshari K.R., Kjaer A., Laustsen C. (2019). Hyperpolarized 13C MRI: Path to Clinical Translation in Oncology. Neoplasia.

[27] Cho A., Lau J.Y.C., Geraghty B.J., Cunningham C.H., Keshari K.R. (2017). Noninvasive interrogation of cancer metabolism with hyperpolarized 13C MRI. J. Nucl. Med..

[28] Malik M., Chaudhary R., Pundir C.S. (2019). An improved enzyme nanoparticles based amperometric pyruvate biosensor for detection of pyruvate in serum. Enzym. Microb. Technol..

[29] Waddington D.E.J., Sarracanie M., Zhang H., Salameh N., Glenn D.R., Rej E., Gaebel T., Boele T., Walsworth R.L., Reilly D.J. (2017). Nanodiamond-enhanced MRI via in situ hyperpolarization. Nat. Commun..

[30] Kostarelos K., Bianco A., Prato M. (2009). Promises, facts and challenges for carbon nanotubes in imaging and therapeutics. Nat. Nanotechnol..

[31] De La Zerda A., Zavaleta C., Keren S., Vaithilingam S., Bodapati S., Liu Z., Levi J., Smith B.R., Ma T.J., Oralkan O. (2008). Carbon nanotubes as photoacoustic molecular imaging agents in living mice. Nat. Nanotechnol..

[32] Welsher K., Liu Z., Sherlock S.P., Robinson J.T., Chen Z., Daranciang D., Dai H. (2009). A route to brightly fluorescent carbon nanotubes for near-infrared imaging in mice. Nat. Nanotechnol..

[33] Garg B., Sung C.H., Ling Y.C. (2015). Graphene-based nanomaterials as molecular imaging agents. Wiley Interdiscip. Rev. Nanomed. Nanobiotechnol..

[34] Nurunnabi M., Khatun Z., Reeck G.R., Lee D.Y., Lee Y.K. (2014). Photoluminescent graphene nanoparticles for cancer phototherapy and imaging. ACS Appl. Mat. Interf..

[35] Cai X., Zhu Q., Zeng Y., Zeng Q., Chen X., Zhan Y. (2019). Manganese oxide nanoparticles as mri contrast agents in tumor multimodal imaging and therapy. Int. J. Nanomed..

[36] Kwiatkowski G., Jähnig F., Steinhauser J., Wespi P., Ernst M., Kozerke S. (2017). Nanometer size silicon particles for hyperpolarized MRI. Sci. Rep..

[37] Yoo S.P., Pineda F., Barrett J.C., Poon C., Tirrell M., Chung E.J. (2016). Gadolinium-functionalized peptide amphiphile micelles for multimodal imaging of atherosclerotic lesions. ACS Omega.

[38] Dong Y.C., Hajfathalian M., Maidment P.S.N., Hsu J.C., Naha P.C., Si-Mohamed S., Breuilly M., Kim J., Chhour P., Douek P. (2019). Effect of gold nanoparticle size on their properties as contrast agents for computed tomography. Sci. Rep..

[39] Silvestri A., Zambelli V., Ferretti A.M., Salerno D., Bellani G., Polito L. (2016). Design of functionalized gold nanoparticle probes for computed tomography imaging. Contrast Media Mol. Imaging.

[40] Nakagawa T., Gonda K., Kamei T., Cong L., Hamada Y., Kitamura N., Tada H., Ishida T., Aimiya T., Furusawa N. (2016). X-ray computed tomography imaging of a tumor with high sensitivity using gold nanoparticles conjugated to a cancer-specific antibody via polyethylene glycol chains on their surface. Sci. Technol. Adv. Mater..

[41] Mok P.L., Leow S.N., Koh A.E.H., Mohd Nizam H.H., Ding S.L.S., Luu C., Ruhaslizan R., Wong H.S., Halim W.H.W.A., Ng M.H. (2017). Micro-computed tomography detection of gold nanoparticle-labelled mesenchymal stem cells in the rat subretinal layer. Int. J. Mol. Sci..

[42] Farooq Aziz A.I., Nazir A., Ahmad I., Bajwa S.Z., Rehman A., Diallo A., Khan W.S. (2017). Novel route synthesis of porous and solid gold nanoparticles for investigating their comparative performance as contrast agent in computed tomography scan and effect on liver and kidney function. Int. J. Nanomed..

[43] Cheheltani R., Ezzibdeh R.M., Chhour P., Pulaparthi K., Kim J., Jurcova M., Hsu J.C., Blundell C., Litt H.I., Ferrari V.A. (2016). Tunable, biodegradable gold nanoparticles as contrast agents for computed tomography and photoacoustic imaging. Biomaterials.

[44] Hainfeld J.F., Ridwan S.M., Stanishevskiy Y., Smilowitz N.R., Davis J., Smilowitz H.M. (2018). Small, long blood half-life iodine nanoparticle for vascular and tumor imaging. Sci. Rep..

[45] Sun N., Zhao L., Zhu J., Li Y., Song N., Xing Y., Qiao W., Huang H., Zhao J. (2019). 131I-labeled polyethylenimine-entrapped gold nanoparticles for targeted tumor Spect/Ct imaging and radionuclide therapy. Int. J. Nanomed..

[46] Kim H., Jang E.J., Kim S.K., Hyung W.J., Choi D.K., Lim S.J., Lim J.S. (2017). Simultaneous sentinel lymph node computed tomography and locoregional chemotherapy for lymph node metastasis in rabbit using an iodine-docetaxel emulsion. Oncotarget.

[47] Badea C.T., Clark D.P., Holbrook M., Srivastava M., Mowery Y., Ghaghada K.B. (2019). Functional imaging of tumor vasculature using iodine and gadolinium-based nanoparticle contrast agents: A comparison of spectral micro-CT using energy integrating and photon counting detectors. Phys. Med. Biol..

[48] Chen J., Yang X.Q., Qin M.Y., Zhang X.S., Xuan Y., Zhao Y.D. (2015). Hybrid nanoprobes of bismuth sulfide nanoparticles and CdSe/ZnS quantum dots for mouse computed tomography/fluorescence dual mode imaging. J. Nanobiotechnol..

[49] Santos B.S., Ferreira M.J. (2019). Positron emission tomography in ischemic heart disease. Rev. Port. Cardiol..

[50] Lee S.B., Lee S.W., Jeong S.Y., Yoon G., Cho S.J., Kim S.K., Lee I.K., Ahn B.C., Lee J., Jeon Y.H. (2017). Engineering of radioiodine-labeled gold core-shell nanoparticles as efficient nuclear medicine imaging agents for trafficking of dendritic cells. ACS Appl. Mater. Interfaces.

[51] Simón M., Jørgensen J.T., Norregaard K., Kjaer A. (2020). 18F-FDG positron emission tomography and diffusion-weighted magnetic resonance imaging for response evaluation of nanoparticle-mediated photothermal therapy. Sci. Rep..

[52] Liu Y., Carpenter A.B., Pirozzi C.J., Yuan H., Waitkus M.S., Zhou Z., Hansen L., Seywald M., Odion R., Greer P.K. (2019). Non-invasive sensitive brain tumor detection using dual-modality bioimaging nanoprobe. Nanotechnology.

[53] Lee S.B., Kumar D., Li Y., Lee I.K., Cho S.J., Kim S.K., Lee S.W., Jeong S.Y., Lee J., Jeon Y.H. (2018). PEGylated crushed gold shell-radiolabeled core nanoballs for in vivo tumor imaging with dual positron emission tomography and Cerenkov luminescent imaging. J. Nanobiotechnol..

[54] Cui L., Xiong C., Zhou M., Shi S., Chow D.S.L., Li C. (2018). Integrin αvβ3-targeted [64 Cu]CuS nanoparticles for PET/CT imaging and photothermal ablation therapy. Bioconjug. Chem..

[55] Woodard P.K., Liu Y., Pressly E.D., Luehmann H.P., Detering L., Sultan D.E., Laforest R., McGrath A.J., Gropler R.J., Hawker C.J. (2016). Design and modular construction of a polymeric nanoparticle for targeted atherosclerosis positron emission tomography imaging: A Story of 25% (64)Cu-CANF-Comb. Pharm. Res..

[56] Kim H.Y., Li R., Ng T., Courties G., Rodell C.B., Prytyskach M., Kohler R.H., Pittet M.J., Nahrendorf M., Weissleder R. (2018). Quantitative imaging of tumor-associated macrophages and their response to therapy using (64)Cu-labeled macrin. ACS Nano.

[57] Wong P., Li L., Chea J., Delgado M.K., Crow D., Poku E., Szpikowska B., Bowles N., Channappa D., Colcher D. (2017). PET imaging of (64)Cu-DOTA-scFv-anti-PSMA lipid nanoparticles (LNPs): Enhanced tumor targeting over anti-PSMA scFv or untargeted LNPs. Nucl. Med. Biol..

[58] Keliher E.J., Ye Y.X., Wojtkiewicz G.R., Aguirre A.D., Tricot B., Senders M.L., Groenen H., Fay F., Perez-Medina C., Calcagno C. (2017). Polyglucose nanoparticles with renal elimination and macrophage avidity facilitate PET imaging in ischaemic heart disease. Nat. Commun..

[59] Sun J., Sun L., Li J., Xu J., Wan Z., Ouyang Z., Liang L., Li S., Zeng D. (2018). A multi-functional polymeric carrier for simultaneous positron emission tomography imaging and combination therapy. Acta Biomater..

[60] Piras A.M., Fabiano A., Sartini S., Zambito Y., Braccini S., Chiellini F., Cataldi A.G., Bartoli F., de la Fuente A., Erba P.A. (2019). pH-responsive carboxymethylcellulose nanoparticles for 68Ga-WBC labelling in PET imaging. Polymers.

[61] Wang X., Yan J., Pan D., Yang R., Wang L., Xu Y., Sheng J., Yue Y., Huang Q., Wang Y. (2018). Polyphenol–poloxamer self-assembled supramolecular nanoparticles for tumor NIRF/PET imaging. Adv. Healthc. Mater..

[62] McDonagh P.R., Sundaresan G., Yang L., Sun M., Mikkelsen R., Zweit J. (2018). Biodistribution and PET imaging of 89-zirconium labeled cerium oxide nanoparticles synthesized with several surface coatings. Nanomedicine.

[63] Chakravarty R., Hong H., Cai W. (2015). Image-guided drug delivery with single-photon emission computed tomography: A review of literature. Curr. Drug Targets.

[64] Si-Mohamed S., Cormode D.P., Bar-Ness D., Sigovan M., Naha P.C., Langlois J.B., Chalabreysse L., Coulon P., Blevis I., Roessl E. (2017). Evaluation of spectral photon counting computed tomography K-edge imaging for determination of gold nanoparticle biodistribution in vivo. Nanoscale.

[65] Estudiante-Mariquez O.J., Rodríguez-Galván A., Ramírez-Hernández D., Contreras-Torres F.F., Medina L.A. (2020). Technetium-radiolabeled mannose-functionalized gold nanoparticles as nanoprobes for sentinel lymph node detection. Molecules.

[66] Hoogendam J.P., Zweemer R.P., Hobbelink M.G.G., Van Den Bosch M.A.A.J., Verheijen R.H.M., Veldhuis W.B. (2016). 99mTc-nanocolloid SPECT/MRI fusion for the selective assessment of nonenlarged sentinel lymph nodes in patients with early-stage cervical cancer. J. Nucl. Med..

[67] Collarino A., Vidal-Sicart S., Perotti G., Valdés Olmos R.A. (2016). The sentinel node approach in gynaecological malignancies. Clin. Transl. Imaging.

[68] Saad Z.Z., Omorphos S., Michopoulou S., Gacinovic S., Malone P., Nigam R., Muneer A., Bomanji J. (2017). Investigating the role of SPECT/CT in dynamic sentinel lymph node biopsy for penile cancers. Eur. J. Nucl. Med. Mol. Imaging.

[69] De Veij Mestdagh P.D., Schreuder W.H., Vogel W.V., Donswijk M.L., Van Werkhoven E., Van Der Wal J.E., Dirven R., Karakullukcu B., Sonke J.J., Van Den Brekel M.W.M. (2019). Mapping of sentinel lymph node drainage using SPECT/CT to tailor elective nodal irradiation in head and neck cancer patients (SUSPECT-2): A single-center prospective trial. BMC Cancer.

[70] De Veij Mestdagh P.D., Schreuder W.H., Vogel W.V., Donswijk M.L., Van Werkhoven E., Van Der Wal J.E., Dirven R., Karakullukcu B., Sonke J.J., Van Den Brekel M.W.M. (2018). An analysis of SPECT/CT non-visualization of sentinel lymph nodes in renal tumors. EJNMMI Res..

[71] Wang Y., Li Y., Wei F., Duan Y. (2017). Optical imaging paves the way for autophagy research. Trends Biotechnol..

[72] Boschi F., de Sanctis F. (2017). Overview of the optical properties of fluorescent nanoparticles for optical imaging. Eur. J. Histochem..

[73] De-La-Cuesta J., González E., Pomposo J.A. (2017). Advances in fluorescent single-chain nanoparticles. Molecules.

[74] Wu W., Yang Y.Q., Yang Y., Yang Y.M., Wang H., Zhang K.Y., Guo L., Ge H.F., Liu J., Feng H. (2019). An organic NIR-II nanofluorophore with aggregation-induced emission characteristics for in vivo fluorescence imaging. Int. J. Nanomed..

[75] Zhong Y., Ma Z., Wang F., Wang X., Yang Y., Liu Y., Zhao X., Li J., Du H., Zhang M. (2019). In vivo molecular imaging for immunotherapy using ultra-bright near-infrared-IIb rare-earth nanoparticles. Nat. Biotechnol..

[76] Bhavane R, Starosolski Z, Stupin I, Ghaghada KB, Annapragada, A (2018). NIR-II fluorescence imaging using indocyanine green nanoparticles. Sci. Rep..

[77] Li C.H., Kuo T.R., Su H.J., Lai W.Y., Yang P.C., Chen J.S., Wang D.Y., Wu Y.C., Chen C.C. (2015). Fluorescence-guided probes of aptamer-targeted gold nanoparticles with computed tomography imaging accesses for in vivo tumor resection. Sci. Rep..

[78] Wallat J.D., Czapar A.E., Wang C., Wen A.M., Wek K.S., Yu X., Steinmetz N.F., Pokorski J.K. (2017). Optical and magnetic resonance imaging using fluorous colloidal nanoparticles. Biomacromolecules.

[79] Kim E.H., Chin G., Rong G., Poskanzer K.E., Clark H.A. (2018). Optical probes for neurobiological sensing and imaging. Acc. Chem. Res..

[80] Napp J., Andrea Markus M., Heck J.G., Dullin C., Möbius W., Gorpas D., Feldmann C., Alves F. (2018). Therapeutic fluorescent hybrid nanoparticles for traceable delivery of glucocorticoids to inflammatory sites. Theranostics.

[81] Wang Y., Xu N., He Y., Wang J., Wang D., Gao Q., Xie S., Li Y., Zhang R., Cai Q. (2019). Loading graphene quantum dots into optical-magneto nanoparticles for real-time tracking in vivo. Materials.

[82] Flak D., Przysiecka Ł., Nowaczyk G., Scheibe B., Kościński M., Jesionowski T., Jurga S. (2018). GQDs-MSNs nanocomposite nanoparticles for simultaneous intracellular drug delivery and fluorescent imaging. J. Nanopart. Res..

[83] Pan Y., Chang T., Marcq G., Liu C., Kiss B., Rouse R., Mach K.E., Cheng Z., Liao J.C. (2017). In vivo biodistribution and toxicity of intravesical administration of quantum dots for optical molecular imaging of bladder cancer. Sci. Rep..

[84] Wang Y.W., Reder N.P., Kang S., Glaser A.K., Liu J.T.C. (2017). Multiplexed optical imaging of tumor-directed nanoparticles: A review of imaging systems and approaches. Nanotheranostics.

[85] Imamura Y., Yamada S., Tsuboi S., Nakane Y., Tsukasaki Y., Komatsuzaki A., Jin T. (2016). Near-infrared emitting PbS quantum dots for in vivo fluorescence imaging of the thrombotic state in septic mouse brain. Molecules.

[86] Bruns O.T., Bischof T.S., Harris D.K., Franke D., Shi Y., Riedemann L., Bartelt A., Jaworski F.B., Carr J.A., Rowlands C.J. (2017). Next-generation in vivo optical imaging with short-wave infrared quantum dots. Nat. Biomed. Eng..

[87] Mintz K.J., Zhou Y., Leblanc R.M. (2019). Recent development of carbon quantum dots regarding their optical properties, photoluminescence mechanism, and core structure. Nanoscale.

[88] Liu X., Braun G.B., Zhong H., Hall D.J., Han W., Qin M., Zhao C., Wang M., She Z.-G., Cao C. (2016). Tumor-targeted multimodal optical imaging with versatile cadmium-free quantum dots. Adv. Funct. Mater..

[89] Ou J., Zhou Z., Chen Z., Tan H. (2019). Optical diagnostic based on functionalized gold nanoparticles. Int. J. Mol. Sci..

[90] Sánchez G.J., Maury P., Stefancikova L., Campion O., Laurent G., Chateau A., Hoch F.B., Boschetti F., Denat F., Pinel S. (2019). Fluorescent radiosensitizing gold nanoparticles. Int. J. Mol. Sci..

[91] Brito H.F., Hölsä J., Laamanen T., Lastusaari M., Malkamäki M., Rodrigues L.C. (2012). Persistent luminescence mechanisms: Human imagination at work. Opt. Mater. Exp..

[92] Lecuyer T., Teston E., Maldiney T., Scherman D., Richard C. (2016). Physico-chemical characterizations of CR doped persistent luminescence nanoparticles. SPIE Bios..

[93] Sharma S.K., Gourier D., Viana B., Maldiney T., Teston E., Scherman D., Richard C. (2015). Persistent luminescence in nanophosphors for long term in-vivo bio-imaging. SPIE Bios..

[94] Ramírez-García G., Gutiérrez-Granados S., Gallegos-Corona M.A., Palma-Tirado L., d’Orlyé F., Varenne A., Mignet N., Richard C., Martínez-Alfaro M. (2017). Long-term toxicological effects of persistent luminescence nanoparticles after intravenous injection in mice. Int. J. Pharm..

[95] Guo R., Lu G., Qin B., Fei B. (2018). Ultrasound imaging technologies for breast cancer detection and management: A review. Ultrasound Med. Biol..

[96] Zhou X., Guo L., Shi D., Duan S., Li J. (2019). Biocompatible chitosan nanobubbles for ultrasound-mediated targeted delivery of doxorubicin. Nanoscale Res. Lett..

[97] Meng M., Gao J., Wu C., Zhou X., Zang X., Lin X., Liu H., Wang C., Su H., Liu K. (2016). Doxorubicin nanobubble for combining ultrasonography and targeted chemotherapy of rabbit with VX2 liver tumor. Tumor Biol..

[98] Cheng B., Bing C., Xi Y., Shah B., Exner A.A., Chopra R. (2019). Influence of nanobubble concentration on blood–brain barrier opening using focused ultrasound under real-time acoustic feedback control. Ultrasound Med. Biol..

[99] Chandan R., Banerjee R. (2018). Pro-apoptotic liposomes-nanobubble conjugate synergistic with paclitaxel: A platform for ultrasound responsive image-guided drug delivery. Sci. Rep..

[100] Thakur S.S., Ward M.S., Popat A., Flemming N.B., Parat M., Barnett N.L., Parekh H.S. (2017). Stably engineered nanobubblesand ultrasound—An effective platform for enhanced macromolecular delivery to representative cells of the retina. PLoS ONE.

[101] Chan M.H., Pan Y.T., Chan Y.C., Hsiao M., Chen C.H., Sun L., Liu R.S. (2018). Nanobubble-embedded inorganic 808 nm excited upconversion nanocomposites for tumor multiple imaging and treatment. Chem. Sci..

[102] Tian Y., Liu Z., Zhang L., Zhang J., Han X., Wang Q., Cheng W. (2018). Apatinib-loaded lipid nanobubbles combined with ultrasound-targeted nanobubble destruction for synergistic treatment of HepG2 cells in vitro. OncoTargets Ther..

[103] Suzuki R., Oda Y., Omata D., Nishiie N., Koshima R., Shiono Y., Sawaguchi Y., Unga J., Naoi T., Negishi Y. (2016). Tumor growth suppression by the combination of nanobubbles and ultrasound. Cancer Sci..

[104] Bhandari P., Novikova G., Goergen C.J., Irudayaraj J. (2018). Ultrasound beam steering of oxygen nanobubbles for enhanced bladder cancer therapy. Sci. Rep..

[105] Wu M., Zhao H., Guo L., Wang Y., Song J., Zhao X., Li C., Hao L., Wang D., Tang J. (2018). Ultrasound-mediated nanobubble destruction (UMND) facilitates the delivery of A10-3.2 aptamer targeted and siRNA-loaded cationic nanobubbles for therapy of prostate cancer. Drug Deliv..

[106] Choi W., Park E.-Y., Jeon S., Kim C. (2018). Clinical photoacoustic imaging platforms. Biomed. Eng. Lett..

[107] Steinberg I., Huland D.M., Vermesh O., Frostig H.E., Tummers W.S., Gambhir S.S. (2019). Photoacoustic clinical imaging. Photoacoustics.

[108] Dumani D.S., Sun I.-C., Emelianov S.Y. (2019). Ultrasound-guided immunofunctional photoacoustic imaging for diagnosis of lymph node metastases. Nanoscale.

[109] Nagaoka R., Tabata T., Yoshizawa S., Umemura S. (2018). Photoacoustics visualization of murine lymph vessels using photoacoustic imaging with contrast agents. Biochem. Pharmacol..

[110] Zhang M., Kim H.S., Jin T., Yi A., Moon W.K. (2016). Ultrasound-guided photoacoustic imaging for the selective detection of EGFR-expressing breast cancer and lymph node metastases. Biomed. Opt. Express.

[111] Li P., Wu Y., Li D., Su X., Luo C., Wang Y., Hu J., Li G., Jiang H., Zhang W. (2018). Seed-mediated synthesis of tunable-aspect-ratio gold nanorods for near-infrared photoacoustic imaging. Nanoscale Res. Lett..

[112] Han S., Bouchard R., Sokolov K.V. (2019). Molecular photoacoustic imaging with ultra-small gold nanoparticles. Biomed. Opt. Express.

[113] Chen Y.-S., Zhao Y., Yoon S.J., Gambhir S.S., Emelianov S. (2019). Miniature gold nanorods for photoacoustic molecular imaging in the second near-infrared optical window. Nat. Nanotechnol..

[114] Sun I.-C., Ahn C.-H., Kim K., Emelianov S. (2019). Photoacoustic imaging of cancer cells with glycol-chitosan-coated gold nanoparticles as contrast agents. J. Biomed. Opt..

[115] Bharathiraja S., Manivasagan P., Bui N.Q., Oh Y.O., Lim I.G., Park S., Oh J. (2016). Cytotoxic induction and photoacoustic imaging of breast cancer cells using astaxanthin-reduced gold nanoparticles. Nanomaterials.

[116] Hartman R.K., Hallam K.A., Donnelly E.M., Emelianov S.Y. (2019). Photoacoustic imaging of gold nanorods in the brain delivered via microbubble-assisted focused ultrasound: A tool for in vivo molecular neuroimaging. Laser Phys. Lett..

[117] Dixon A.J., Hu S., Klibanov A.L., Hossack J.A. (2015). Oscillatory dynamics and in vivo photoacoustic imaging performance of plasmonic nanoparticle-coated microbubbles. Small.

[118] Augustine S., Singh J., Srivastava M., Sharma M., Das A., Malhotra B.D. (2017). Recent advances in carbon based nanosystems for cancer theranostics. Biomater. Sci..

[119] Xie L., Wang G., Zhou H., Zhang F., Guo Z., Liu C., Zhang X., Zhu L. (2016). Functional long circulating single walled carbon nanotubes for fluorescent/photoacoustic imaging-guided enhanced phototherapy. Biomaterials.

[120] Fathi P., Knox H.J., Sar D., Tripathi I., Ostadhossein F., Misra S.K., Esc M.B., Chan J., Pan D. (2019). Biodegradable biliverdin nanoparticles for efficient photoacoustic imaging. ACS Nano.

[121] Köker T., Tang N., Tian C., Tian C., Zhang W., Wang X., Martel R., Pinaud F. (2018). Cellular imaging by targeted assembly of hot-spot SERS and photoacoustic nanoprobes using split-fluorescent protein scaffolds. Nat. Commun..

[122] Tan J.W.Y., Murashov M.D., Rosania G.R., Wang X. (2019). Photoacoustic imaging of clofazimine hydrochloride nanoparticle accumulation in cancerous vs normal prostates. PLoS ONE.

[123] Swider E., Daoudi K., Staal A.H.J., Koshkina O., Koen van Riessen N., van Dinther E., de Vries I.J.M., de Korte C.L., Srinivas M. (2018). Clinically-applicable perfluorocarbon-loaded nanoparticles for in vivo photoacoustic,19f magnetic resonance and fluorescent imaging. Nanotheranostics.

[124] Biffi S., Petrizza L., Garrovo C., Rampazzo E., Andolfi L., Giustetto P., Nikolov I., Kurdi G., Danailov M.B., Zauli G. (2016). Multimodal near-infrared-emitting PluS Silica nanoparticles with fluorescent, photoacoustic, and photothermal capabilities. Int. J. Nanomed..

[125] Harrington W.N., Haji M.R., Galanzha E.I., Nedosekin D.A., Nima Z.A., Watanabe F., Ghosh A., Biris A.S., Zharov V.P. (2016). Photoswitchable non-fluorescent thermochromic dye-nanoparticle hybrid probes. Sci. Rep..

[126] Wang Y., Shang W., Niu M., Tian J., Xu K. (2019). Hypoxia-active nanoparticles used in tumor theranostic. Int. J. Nanomed..

[127] Hirschberg H., Madsen S.J. (2017). Cell mediated photothermal therapy of brain tumors. J. Neuroimmune Pharmacol. Off. J. Soc. Neuroimmune Pharmacol..

[128] Vines J.B., Yoon J.H., Ryu N.E., Lim D.J., Park H. (2019). Gold nanoparticles for photothermal cancer therapy. Front. Chem..

[129] Simón M., Norregaard K., Jørgensen J.T., Oddershede L.B., Kjaer A. (2019). Fractionated photothermal therapy in a murine tumor model: Comparison with single dose. Int. J. Nanomed..

[130] Riley R.S., Day E.S. (2017). Gold nanoparticle-mediated photothermal therapy: Applications and opportunities for multimodal cancer treatment. Wiley Interdiscip. Rev. Nanomed. Nanobiotechnol..

[131] Jørgensen J.T., Norregaard K., Tian P., Bendix P.M., Kjaer A., Oddershede L.B. (2016). Single particle and PET-based platform for identifying optimal plasmonic nano-heaters for photothermal cancer therapy. Sci. Rep..

[132] Estelrich J., Antònia Busquets M. (2018). Iron oxide nanoparticles in photothermal therapy. Molecules.

[133] Vines J.B., Lim D.J., Park H. (2018). Contemporary polymer-based nanoparticle systems for photothermal therapy. Polymers.

[134] Wu M., Le W., Mei T., Wang Y., Chen B., Liu Z., Xue C. (2019). Cell membrane camouflaged nanoparticles: A new biomimetic platform for cancer photothermal therapy. Int. J. Nanomed..

[135] Mokoena D.R., George B.P., Abrahamse H. (2019). Enhancing breast cancer treatment using a combination of cannabidiol and gold nanoparticles for photodynamic therapy. Int. J. Mol. Sci..

[136] Bretin L., Pinon A., Bouramtane S., Ouk C., Richard L., Perrin M., Chaunavel A., Carrion C. (2019). Photodynamic therapy activity of new human colorectal cancer. Cancers.

[137] Abrahamse H., Hamblin M.R. (2016). New photosensitizers for photodynamic therapy. Biochem. J..

[138] Honors C.N., Kruger C.A., Abrahamse H. (2018). Photodynamic therapy for metastatic melanoma treatment: A review. Technol. Cancer Res. Treat..

[139] Yu Z., Zhou P., Pan W., Li N., Tang B. (2018). A biomimetic nanoreactor for synergistic chemiexcited photodynamic therapy and starvation therapy against tumor metastasis. Nat. Commun..

[140] Zhu T., Shi L., Yu C., Dong Y., Qiu F., Shen L., Qian Q., Zhou G., Zhu X. (2019). Ferroptosis promotes photodynamic therapy: Supramolecular photosensitizer-inducer nanodrug for enhanced cancer treatment. Theranostics.

[141] Liyanage P.Y., Hettiarachchi S.D., Zhou Y., Ouhtit A., Seven E.S., Oztan C.Y., Celik E., Leblanc R.M. (2019). Nanoparticle-mediated targeted drug delivery for breast cancer treatment. Biochim. Biophys. Acta Rev. Cancer.

[142] Lee M.S., Dees E.C., Wang A.Z. (2017). Nanoparticle-delivered chemotherapy: Old drugs in new packages. Oncology.

[143] Press D. (2018). Application of active targeting nanoparticle delivery system for chemotherapeutic drugs and traditional/herbal medicines in cancer therapy: A systematic review. Int. J. Nanomed..

[144] Pelt J., Busatto S., Ferrari M., Thompson E.A., Mody K., Wolfram J. (2018). Chloroquine and nanoparticle drug delivery: A promising combination. Pharmacol. Ther..

[145] Singh P., Pandit S., Mokkapati V.R.S.S., Garg A., Ravikumar V., Mijakovic I. (2018). Gold nanoparticles in diagnostics and therapeutics for human cancer. Int. J. Mol. Sci..

[146] Duan X., He C., Kron S.J., Lin W. (2016). Nanoparticle formulations of cisplatin for cancer therapy. Wiley Interdiscip. Rev. Nanomed. Nanobiotechnol..

[147] Meng F., Han N., Yeo Y. (2017). Organic nanoparticle systems for spatiotemporal control of multimodal chemotherapy. Expert Opin. Drug Deliv..

[148] Fisusi F.A., Akala E.O. (2019). Drug combinations in breast cancer therapy. Pharm. Nanotechnol..

[149] Song M., Liu T., Shi C., Zhang X., Chen X. (2016). Bioconjugated manganese dioxide nanoparticles enhance chemotherapy response by priming tumor-associated macrophages toward M1-like phenotype and attenuating tumor hypoxia. ACS Nano.

[150] Wang B., Zhang W., Zhou X., Liu M., Hou X., Cheng Z., Chen D. (2019). Development of dual-targeted nano-dandelion based on an oligomeric hyaluronic acid polymer targeting tumor-associated macrophages for combination therapy of non-small cell lung cancer. Drug Deliv..

[151] Mangal S., Gao W., Li T., Zhou Q.T. (2017). Pulmonary delivery of nanoparticle chemotherapy for the treatment of lung cancers: Challenges and opportunities. Acta Pharmacol. Sin..

[152] Saleh T., Shojaosadati S.A. (2016). Multifunctional nanoparticles for cancer immunotherapy. Hum. Vaccines Immunother..

[153] Riley R.S., June C.H., Langer R., Mitchell M.J. (2019). Delivery technologies for cancer immunotherapy. Nat. Rev. Drug Discov..

[154] Wen R., Umeano A.C., Kou Y., Xu J., Farooqi A.A. (2019). Nanoparticle systems for cancer vaccine. Nanomedicine.

[155] Park W., Heo Y.-J., Han D.K. (2018). New opportunities for nanoparticles in cancer immunotherapy. Biomater. Res..

[156] Sau S., Alsaab H.O., Bhise K., Alzhrani R., Nabil G., Iyer A.K. (2018). Multifunctional nanoparticles for cancer immunotherapy: A groundbreaking approach for reprogramming malfunctioned tumor environment. J. Control. Release.

[157] Surendran S.P., Moon M.J., Park R., Jeong Y.Y. (2018). Bioactive nanoparticles for cancer immunotherapy. Int. J. Mol. Sci..

[158] Wilson D.R., Sen R., Sunshine J.C., Pardoll D.M., Green J.J., Kim Y.J. (2018). Biodegradable STING agonist nanoparticles for enhanced cancer immunotherapy. Nanomedicine.

[159] Shoeb E., Hefferon K. (2019). Future of cancer immunotherapy using plant virus-based nanoparticles. Future Sci. OA.

[160] Evans E.R., Bugga P., Asthana V., Drezek R. (2018). Metallic nanoparticles for cancer immunotherapy. Mater. Today.

[161] Jia Y., Omri A., Krishnan L., McCluskie M.J. (2017). Potential applications of nanoparticles in cancer immunotherapy. Hum. Vaccines Immunother..

[162] Rodell C.B., Arlauckas S.P., Cuccarese M.F., Garris C.S., Li R., Ahmed M.S., Kohler R.H., Pittet M.J., Weissleder R. (2018). TLR7/8-agonist-loaded nanoparticles promote the polarization of tumour-associated macrophages to enhance cancer immunotherapy. Nat. Biomed. Eng..

[163] Alipoor S.D., Mortaz E., Varahram M., Movassaghi M., Kraneveld A.D., Garssen J., Adcock I.M. (2018). The potential biomarkers and immunological effects of tumor-derived exosomes in lung cancer. Front. Immunol..

[164] Ballinger J.R. (2018). Theranostics and precision medicine special feature: Review Article Theranostic radiopharmaceuticals: Established agents in current use. Br. J. Radiol..

[165] Yuan H., Wilks M.Q., Maschmeyer R., Normandin M.D., Josephson L., Fakhri G.E. (2020). Original research a radio-nano-platform for T1/T2 dual-mode PET-MR imaging. Int. J. Nanomed..

[166] Evertsson M., Kjellman P., Cinthio M., Andersson R., Tran T.A., In’t Zandt R., Grafström G., Toftevall H., Fredriksson S., Ingvar C. (2017). Combined magnetomotive ultrasound, PET/CT, and MR imaging of 68Ga-labelled superparamagnetic iron oxide nanoparticles in rat sentinel lymph nodes in vivo. Sci. Rep..

[167] Shin S.H., Park S.H., Kang S.H., Kim S.W., Kim M., Kim D. (2017). Fluorine-19 magnetic resonance imaging and positron emission tomography of tumor-associated macrophages and tumor metabolism. Contrast Media Mol. Imaging.

[168] Thomas E. (2017). Ga-radiolabeled AGuIX nanoparticles as dual-modality imaging agents for PET/MRI-guided radiotherapy. Nanomedicine.

[169] Martelli S., Chow J.C.L. (2020). Dose enhancement for the flattening-filter-free and flattening-filter photon beams in nanoparticle-enhanced radiotherapy: A monte carlo phantom study. Nanomaterials.

[170] Mututantri-Bastiyange D., Chow J.C.L. (2020). Imaging dose of cone-beam computed tomography in nanoparticle-enhanced image-guided radiotherapy: A Monte Carlo phantom study. AIMS Bioeng..

[171] Chow J.C.L., Chan M.F. (2018). Monte carlo nanodosimetry in gold nanoparticle-enhanced radiotherapy. Recent Advancements and Applications Applications in Dosimetry.

[172] Detappe A., Thomas E., Tibbitt M.W., Kunjachan S., Zavidij O., Parnandi N., Reznichenko E., Lux F., Tillement O., Berbeco R. (2017). Ultrasmall silica-based bismuth gadolinium nanoparticles for dual magnetic resonance-computed tomography image guided radiotherapy. Nano Lett..

[173] Chu C.H., Cheng S.H., Chen N.T., Liao W.N., Lo L.W. (2019). Microwave-synthesized platinum-embedded mesoporous silica nanoparticles as dual-modality contrast agents: Computed tomography and optical imaging. Int. J. Mol. Sci..

[174] Nguyen Pham T.H., Lengkeek N.A., Greguric I., Kim B.J., Pellegrini P.A., Bickley S.A., Tanudji M.R., Jones S.K., Hawkett B.S., Pham B.T.T. (2017). Tunable and noncytotoxic PET/SPECT-MRI multimodality imaging probes using colloidally stable ligand-free superparamagnetic iron oxide nanoparticles. Int. J. Nanomed..

[175] Silva F., Paulo A., Pallier A., Même S., Tóth É., Gano L., Marques F., Geraldes C.F.G.C., Castro M.M.C.A., Cardoso A.M. (2020). Dual imaging gold nanoplatforms for targeted radiotheranostics. Materials.

[176] Chen X., Zhou H., Li X., Duan N., Hu S., Liu Y., Yue Y., Song L., Zhang Y., Li D. (2018). Plectin-1 targeted dual-modality nanoparticles for pancreatic cancer imaging. EBioMedicine.

[177] Zheng Y., Zhang H., Hu Y., Bai L., Xue J. (2018). MnO nanoparticles with potential application in magnetic resonance imaging and drug delivery for myocardial infarction. Int. J. Nanomed..

[178] Gonawala S., Ali M.M. (2017). Application of dendrimer-based nanoparticles in glioma imaging. J. Nanomed. Nanotechnol..

[179] Zhou L., Jing Y., Liu Y., Liu Z., Gao D., Chen H., Song W., Wang T., Fang X., Qin W. (2018). Mesoporous carbon nanospheres as a multifunctional carrier for cancer theranostics. Theranostics.

[180] Zhang Y., Tang Z., Wu Y. (2018). The dual-mode imaging of nanogold-labeled cells by photoacoustic microscopy and fluorescence optical microscopy. Technol. Cancer Res. Treat..

[181] Yang Z., Song J., Dai Y., Chen J., Wang F., Lin L., Liu Y., Zhang F., Yu G., Zhou Z. (2017). Theranostics self-assembly of semiconducting-plasmonic gold nanoparticles with enhanced optical property for photoacoustic imaging and photothermal therapy. Theranostics.

[182] Knights O.B., McLaughlan J.R. (2018). Gold nanorods for light-based lung cancer theranostics. Int. J. Mol. Sci..

[183] Song J., Yang X., Jacobson O., Lin L., Huang P., Niu G., Ma Q., Chen X. (2015). Sequential drug release and enhanced photothermal and photoacoustic effect of hybrid reduced graphene oxide-loaded ultrasmall gold nanorod vesicles for cancer therapy. ACS Nano.

[184] Lu S.T., Xu D., Liao R.F., Luo J.Z., Liu Y.H., Qi Z.H., Zhang C.J., Ye N.L., Wu B., Xu H.B. (2019). Single-component bismuth nanoparticles as a theranostic agent for multimodal imaging-guided glioma therapy. Comput. Struct. Biotechnol. J..

[185] Siregar S., Oktamuliani S., Saijo Y. (2018). A theoretical model of laser heating carbon nanotubes. Nanomaterials.

[186] Chow J.C.L., Martinez L.M.T., Kharissova O.V., Kharisov B.I. (2017). Application of nanoparticle materials in radiation therapy. Handbook of Ecomaterials.

[187] Chow J.C.L., Grumezescu A.M. (2016). Photon and electron interactions with gold nanoparticles: A Monte Carlo study on gold nanoparticle-enhanced radiotherapy. Nanobiomaterials in Medical Imaging: Applications of Nanobiomaterials.

[188] Chow J.C.L., Kharissova O.V., Torres-Martínez L.M., Kharisov B.I. (2020). Recent progress of gold nanomaterials in cancer therapy. Handbook of Nanomaterials and Nanocomposites for Energy and Environmental Applications.

[189] He C., Chow J.C. (2016). Gold nanoparticle DNA damage in radiotherapy: A Monte Carlo study. AIMS Bioeng..

[190] Wang, Yanjie, Strohm, E (2016). M.; Sun, Y.; Wang, Z.; Zheng, Y.; Wang, Z.; Kolios, M.C. Biodegradable polymeric nanoparticles containing gold nanoparticles and Paclitaxel for cancer imaging and drug delivery using photoacoustic methods. Biomed. Opt. Express.

[191] Ashton J.R., Castle K.D., Qi Y., Kirsch D.G., West J.L., Badea C.T. (2018). Dual-energy CT imaging of tumor liposome delivery after gold nanoparticle-augmented radiotherapy. Theranostics.

[192] Siddique S., Chow J.C. (2020). Gold nanoparticles for drug delivery and cancer therapy. Appl. Sci..

[193] Du F., Lou J., Jiang R., Fang Z., Zhao X., Niu Y., Zou S., Zhang M., Gong A., Wu C. (2017). Hyaluronic acid-functionalized bismuth oxide nanoparticles for computed tomography imaging-guided radiotherapy of tumor. Int. J. Nanomed..

[194] Li W., Hou W., Guo X., Luo L., Li Q., Zhu C., Yang J., Zhu J., Du Y., You J. (2018). Temperature-controlled, phase-transition ultrasound imaging-guided photothermal-chemotherapy triggered by NIR light. Theranostics.

[195] Liu Z., Zhang J., Tian Y., Zhang L., Han X., Wang Q., Cheng W. (2018). Targeted delivery of reduced graphene oxide nanosheets using multifunctional ultrasound nanobubbles for visualization and enhanced photothermal therapy. Int. J. Nanomed..

[196] Prabhakar A., Banerjee R. (2019). Nanobubble liposome complexes for diagnostic imaging and ultrasound-triggered drug delivery in cancers: A theranostic approach. ACS Omega.

[197] Chen F., Hableel G., Zhao E.R., Jokerst J.V. (2018). Multifunctional nanomedicine with silica: Role of silica in nanoparticles for theranostic, imaging, and drug monitoring. J. Colloid Interface Sci..

[198] Yao H.C., Su L., Zeng M., Cao L., Zhao W.W., Chen C.Q., Du B., Zhou J. (2016). Construction of magnetic-carbon-quantum-dots-probe-labeled apoferritin nanocages for bioimaging and targeted therapy. Int. J. Nanomed..

[199] Saowalak K., Titipun T., Somchai T., Chalermchai P. (2018). Iron(III)-tannic molecular nanoparticles enhance autophagy effect and T1 MRI contrast in liver cell lines. Sci. Rep..

[200] Li L.H., Lu Y., Jiang C.Y., Zhu Y., Yang X.F., Hu X.M., Lin Z.F., Zhang Y., Peng M.Y., Xia H. (2018). Actively targeted deep tissue imaging and photothermal-chemo therapy of breast cancer by antibody-functionalized drug-loaded X-ray-responsive bismuth Sulfide@Mesoporous silica core-shell nanoparticles. Adv. Funct. Mater..

[201] Xu C., Feng Q., Yang H., Wang G., Huang L., Bai Q., Zhang C., Wang Y., Chen Y., Cheng Q. (2018). A light-triggered mesenchymal stem cell delivery system for photoacoustic imaging and chemo-photothermal therapy of triple negative breast cancer. Adv. Sci..

[202] Li G., Chen Y., Zhang L., Zhang M., Li S., Li L., Wang T., Wang C. (2018). Facile approach to synthesize gold Nanorod@Polyacrylic acid/calcium phosphate yolk–shell nanoparticles for dual-mode imaging and pH/NIR-responsive drug delivery. Nano-Micro Lett.

[203] Yang Y., Zhang J., Xia F., Zhang C., Qian Q., Zhi X., Yue C., Sun R., Cheng S., Fang S. (2016). Human CIK cells loaded with Au nanorods as a theranostic platform for targeted photoacoustic imaging and enhanced immunotherapy and photothermal therapy. Nanoscale Res. Lett..

[204] Li K., Nejadnik H. (2017). Daldrup-Link HE. Next-generation superparamagnetic iron oxide nanoparticles for cancer theranostics. Drug Discov. Today.

[205] Gurunathan S., Kang M.H., Qasim M., Kim J.H. (2018). Nanoparticle-mediated combination therapy: Two-in-one approach for cancer. Int. J. Mol. Sci..

[206] Zhang A., Pan S., Zhang Y., Chang J., Cheng J., Huang Z., Li T., Zhang C., De La Fuentea J.M., Zhang Q. (2019). Carbon-gold hybrid nanoprobes for real-time imaging, photothermal/photodynamic and nanozyme oxidative therapy. Theranostics.

[207] Miao W., Kim H., Gujrati V., Kim J.Y., Jon H., Lee Y., Choi M., Kim J., Lee S., Lee D.Y. (2016). Photo-decomposable organic nanoparticles for combined tumor optical imaging and multiple phototherapies. Theranostics.

[208] Yildiz T., Gu R., Zauscher S., Betancourt T. (2018). Doxorubicin-loaded protease-activated near-infrared fluorescent polymeric nanoparticles for imaging and therapy of cancer. Int. J. Nanomed..

[209] Nam J., Son S., Ochyl L.J., Kuai R., Schwendeman A., Moon J.J. (2018). Chemo-photothermal therapy combination elicits anti-tumor immunity against advanced metastatic cancer. Nat. Commun..

[210] Zhang X., Li Y., Wei M., Liu C., Yang J. (2019). Cetuximab-modified silica nanoparticle loaded with ICG for tumor-targeted combinational therapy of breast cancer. Drug Deliv..

[211] Locatelli E., Li Y., Monaco I., Guo W., Maturi M., Menichetti L., Armanetti P., Martin R.C., Franchini M.C. (2019). A novel theranostic gold nanorods- and adriamycin-loaded micelle for EpCA M targeting, laser ablation, and photoacoustic imaging of cancer stem cells in hepatocellular carcinoma. Int. J. Nanomed..

[212] Ramanathan S., Archunan G., Sivakumar M., Selvan S.T., Fred A.L., Kumar S., Gulyás B., Padmanabhan P. (2018). Theranostic applications of nanoparticles in neurodegenerative disorders. Int. J. Nanomed..

[213] Ge X., Fu Q., Su L., Li Z., Zhang W., Chen T., Yang H., Song J. (2020). Light-activated gold nanorod vesicles with NIR-II fluorescence and photoacoustic imaging performances for cancer theranostics. Theranostics.

[214] He C., Duan X., Guo N., Chan C., Poon C., Weichselbaum R.R., Lin W. (2016). Core-shell nanoscale coordination polymers combine chemotherapy and photodynamic therapy to potentiate checkpoint blockade cancer immunotherapy. Nat. Commun..

[215] Chen W., Qin M., Chen X., Wang Q., Zhang Z., Sun X. (2018). Combining photothermal therapy and immunotherapy against melanoma by polydopamine-coated Al_2_O_3_ nanoparticles. Theranostics.

[216] Hou X., Tao Y., Pang Y., Li X., Jiang G., Liu Y. (2018). Nanoparticle-based photothermal and photodynamic immunotherapy for tumor treatment. Int. J. Cancer.

[217] Chen Q., Xu L., Liang C., Wang C., Peng R., Liu Z. (2016). Photothermal therapy with immune-adjuvant nanoparticles together with checkpoint blockade for effective cancer immunotherapy. Nat. Commun..

[218] Mu Q., Wang H., Zhang M. (2017). Nanoparticles for imaging and treatment of metastatic breast cancer. Expert Opin. Drug Deliv..

[219] Zhang J., Zhao T., Han F., Hu Y., Li Y. (2019). Photothermal and gene therapy combined with immunotherapy to gastric cancer by the gold nanoshell-based system. J. Nanobiotechnol..

[220] Li W., Yang J., Luo L., Jiang M., Qin B., Yin H., Zhu C., Yuan X., Zhang J., Luo Z. (2019). Targeting photodynamic and photothermal therapy to the endoplasmic reticulum enhances immunogenic cancer cell death. Nat. Commun..

[221] Min K.H., Kim Y.H., Wang Z., Kim J., Kim J.S., Kim S.H., Kim K., Kwon I.C., Kiesewetter D.O., Chen X. (2017). Theranostics engineered Zn(II)-dipicolylamine-gold nanorod provides effective prostate cancer treatment by combining siRNA delivery and photothermal therapy. Theranostics.

